# A global checklist of the 932 fruit fly species in the tribe Dacini (Diptera, Tephritidae)

**DOI:** 10.3897/zookeys.730.21786

**Published:** 2018-01-17

**Authors:** Camiel Doorenweerd, Luc Leblanc, Allen L. Norrbom, Michael San Jose, Daniel Rubinoff

**Affiliations:** 1 University of Hawaii, Department of Plant and Environmental Protection Services, 3050 Maile Way, Honolulu, Hawaii, 96822-2231, USA; 2 University of Idaho, Department of Entomology, Plant Pathology and Nematology, 875 Perimeter Drive, MS2329, Moscow, Idaho, 83844-2329, USA; 3 Systematic Entomology Laboratory, ARS, USDA, c/o Smithsonian Institution, P.O. Box 37012, MRC 168, Washington, DC 20013-7012, USA

**Keywords:** global, pest, cryptic, *Bactrocera*, *Zeugodacus*, *Dacus*

## Abstract

The correct application of the scientific names of species is neither easy nor trivial. Mistakes can lead to the wrong interpretation of research results or, when pest species are involved, inappropriate regulations and limits on trade, and possibly quarantine failures that permit the invasion of new pest species. Names are particularly challenging to manage when groups of organisms encompass a large number of species, when different workers employ different philosophical views, or when species are in a state of taxonomic flux. The fruit fly tribe Dacini is a species-rich taxon within Tephritidae and contains around a fifth of all known species in the family. About 10% of the 932 currently recognized species are pests of commercial fruits and vegetables, precipitating quarantines and trade embargos. Authoritative species lists consist largely of scattered regional treatments and outdated online resources. The checklist presented here is the first global overview of valid species names for the Dacini in almost two decades, and includes new lure records. By publishing this list both in paper and digitally, we aim to provide a resource for those studying fruit flies as well as researchers studying components of their impact on agriculture. The list is largely a consolidation of previous works, but following the results from recent phylogenetic work, we transfer one subgenus and eight species to different genera: members of the Bactrocera subgenus Javadacus Hardy, considered to belong to the *Zeugodacus* group of subgenera, are transferred to genus *Zeugodacus*; *Bactrocera pseudocucurbitae* White, 1999, **stat. rev.**, is transferred back to *Bactrocera* from *Zeugodacus*; *Zeugodacus arisanicus* Shiraki, 1933, **stat. rev.**, is transferred back to *Zeugodacus* from *Bactrocera*; and *Z. brevipunctatus* (David & Hancock, 2017), **comb. n.**; *Z. javanensis* (Perkins, 1938), **comb. n.**; *Z. montanus* (Hardy, 1983), **comb. n.**; *Z. papuaensis* (Malloch, 1939), **comb. n.**; *Z. scutellarius* (Bezzi, 1916), **comb. n.**; *Z. semisurstyli* (Drew & Romig, 2013), **comb. n.**; and *Z. trilineatus* (Hardy, 1955), **comb. n.** are transferred from *Bactrocera* to *Zeugodacus*.

## Introduction

Despite the current ‘phylogenomic’ age and the generation of large amounts of data on relatively few, selected, organisms, discovering and classifying new species is an ongoing endeavor of basic science that is far from complete (Zhang 2011). Major challenges to advance taxonomic work lie, among others, in the correct application of scientific species names, which in turn depends on the availability of accurate reference databases. Global initiatives to provide reference lists of species names (e.g., Roskov et al. 2017) all include major gaps that can only be filled by taxonomic specialists. Some groups of organisms are particularly challenging to manage because of the number of species they encompass, conceptual differences between workers, or the existence of unresolved problems with species identities or concepts themselves. Simultaneously, those same groups will likely benefit the most from an authoritative overview.

**Figure 1.**
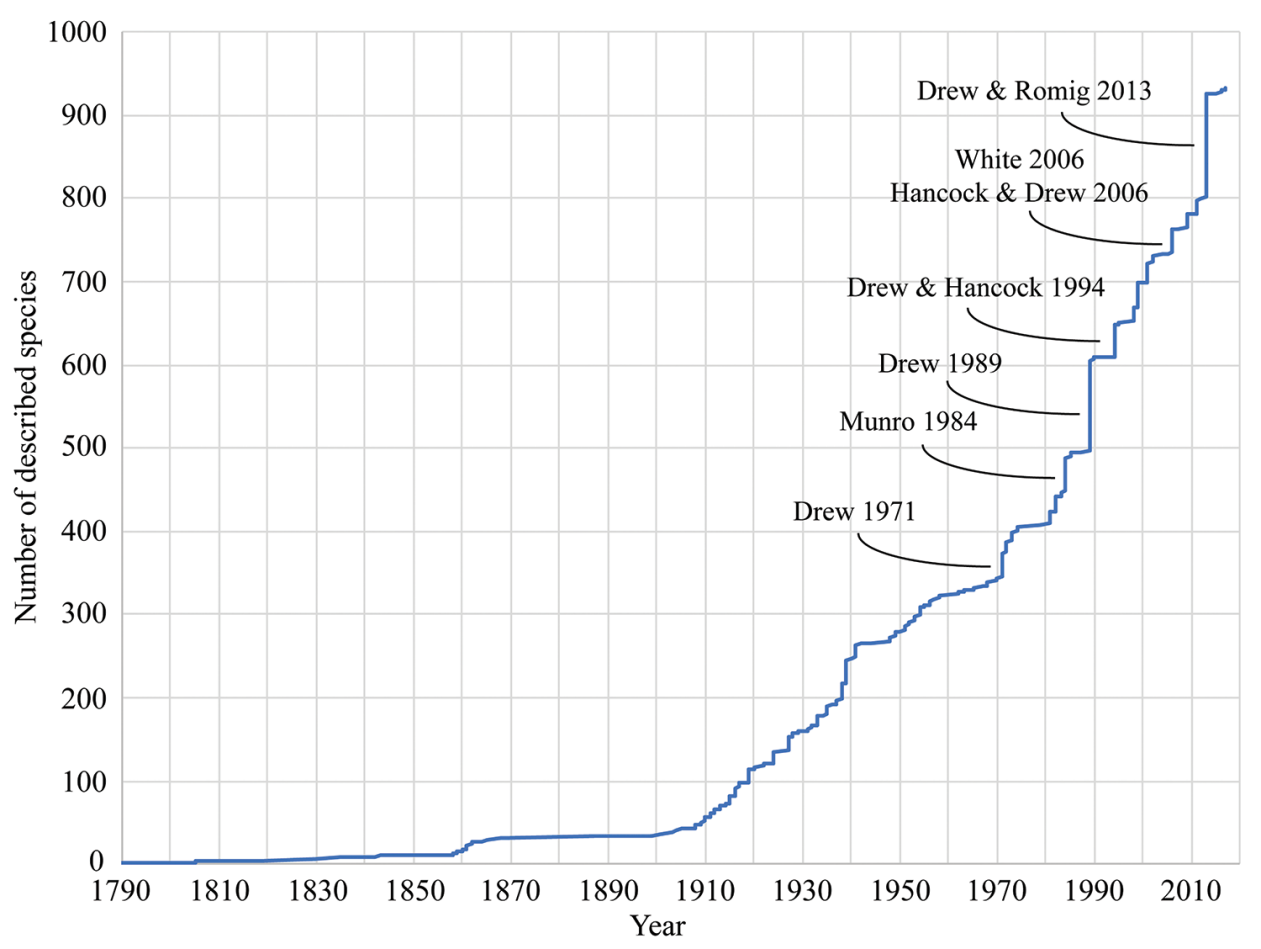
Accumulation of described species in Dacini through time, with publications that featured large numbers of newly described species indicated at their respective moment. The first species was described in 1790, but during the past fifty years the number of recognized [or known] species has more than doubled to reach the current 932.

The fruit fly tribe Dacini is a species-rich radiation within Tephritidae and contains around a fifth of all known species in the family (Norrbom et al. 1999, Pape et al. 2011, Schutze et al. 2017). All Dacini members are frugivorous or florivorous and about 10% of the 932 currently recognized species are pests of commercial fruits and vegetables (Fletcher 1987, White and Elson-Harris 1992, Vargas et al. 2015, Freidberg et al. 2017). Among these are some of the world’s economically most important pests, such as the widely introduced oriental fruit fly, *Bactrocera dorsalis* (Hendel, 1912), carambola fruit fly *Bactrocera carambolae* Drew & Hancock, 1994, and the melon fly, *Zeugodacus cucurbitae* (Coquillett, 1899) (De Meyer et al. 2015, Ekesi et al. 2016). The tribe as a whole has received considerable taxonomic attention and new species are continuously being discovered (Fig. 1; Leblanc et al. 2015a, David et al. 2016, 2017). Dacini flies are phenotypically very similar and therefore also one of the most difficult groups of Tephritidae to identify to species-level. Whereas many Tephritidae can be identified from their intricate wing patterns, which are commonly thought to have evolved to deter predators (such as Salticidae jumping spiders [Whitman et al. 1988]), for mating rituals, or territorial behavior, the wings of most Dacini are clear with only a costal band and, usually, an anal streak. The adult chaetotaxy is a set of characters that is usually of value in dipteran species identification, but in Dacini the number of setae is reduced and similar configurations may often be homoplaseous (Hardy 1955, Hancock and Drew 2015). Their body colors, various combinations of black and yellow to red, are commonly thought to have resulted from wasp mimicry and may be under selective pressure (White 2006). Diagnostic body color patterns used to separate species are further confounded by considerable intraspecific variation (Leblanc et al. 2015b). The combination of these factors has resulted in a long history of unstable classification and even though molecular phylogenetic studies are now reaching a general consensus, this has not fully trickled down to the nomenclatural level.

Dacini is a tropical and subtropical evolutionary radiation of flies with centers of diversity in Southeast Asia and Sub-Saharan Africa. Early molecular phylogenetic studies focused on pest species, often of a particular region, leading to biased results on the relationships between species that may not accurately reflect monophyletic origins or sister-group assignments (Smith et al. 2003, Nakahara and Muraji 2008, Virgilio et al. 2015). With phylogenetic studies expanding their scope beyond the pest species and increased use of molecular data, it became clear that the initial morphology-based classifications had to be revised and, in particular, the large genus *Bactrocera* had to be split into *Bactrocera* and *Zeugodacus* because the latter is more closely related to *Dacus* (Krosch et al. 2012, Virgilio et al. 2015, San Jose et al. 2018 in press, Dupuis et al. 2017). Following the most recent results, there are currently four genera in Dacini: *Monacrostichus* Bezzi, *Dacus* Fabricius, *Bactrocera* Macquart and *Zeugodacus* Hendel (De Meyer et al. 2015, Virgilio et al. 2015, Freidberg et al. 2017), although some authors (e.g., Drew and Romig 2013, 2016, Hancock and Drew 2016) continue to include *Zeugodacus* within *Bactrocera*. *Ichneumonopsis* Hardy is now placed in Gastrozonini (Norrbom et al. 1999, Freidberg et al. 2017). Aside from shifts in generic assignments, taxa have been variably assigned to species complexes, species groups, subgenera and species-complex groups to provide some additional systematic structure, primarily for the purpose of identification keys (Clarke et al. 2005, White 2006, Drew and Romig 2013). These intermediate taxonomic ranks are mostly groups of convenience defined by unique combinations of characters rather than by synapomorphic characters. The largest and most intesively studied is the *Bactrocera dorsalis* complex with 88 species; the group that, incidentally, also holds the largest number of pest species. This complex, like most others, is not monophyletic (Leblanc et al. 2015b, Virgilio et al. 2015, San Jose et al. 2018 in press) and there has been synonymy of several significant pest species, such as *B. papayae* Drew & Hancock, 1994, and *B. invadens* Drew, Tsuruta & White, 2005 with *B. dorsalis* (San Jose et al. 2013, Schutze et al. 2015a, 2015b). To facilitate communication and progress of our understanding of the group, a reliable taxonomic starting point is badly needed and will enable further studies into the taxonomy and systematics of the tribe.

The most recently published catalogs that covered Dacini globally are now almost two decades old (Norrbom et al. 1999, Norrbom 2004) and scattered regional treatments and keys currently comprise the largest body of references for Dacini. For Southeast Asia, there is a relatively recent two-part work including a revision (Drew and Romig 2013) and the accompanying keys that followed (Drew and Romig 2016). These books have incorporated the previous keys for the *B. dorsalis* complex of Drew and Hancock (1994), but they did not adopt the latest results from a series of molecular phylogenetic works, including the split of *Bactrocera* into *Bactrocera* and *Zeugodacus*. For other regions, all treatments are older with increased confusion due to differing morphological terminology, species designations, and assignments. For Africa, the most recent works are two treatments from 2006 (Hancock and Drew 2006, White 2006), and for Australasia there is a treatment from 1989 (Drew 1989), including keys, a proposed subgeneric classification, and revisions for the species in the region. As of 2017, the Drew and Romig books on the Asian fauna are in print and available for sale, and the other works are available digitally online and provide important catalogue references. Online resources that aim to provide up-to-date species checklists such as Systema Dipterorum (Pape and Thompson 2013), the Catalogue of Life (Roskov et al. 2017), or the pest-oriented database of the Centre for Agriculture and Biosciences (CABI 2017) are outdated and have not been able to combine the regional treatments appropriately. Valid and invalid names can be verified using the Tephritidae Databases on the COFFHI website (https://coffhi.cphst.org/), but it was primarily designed for host plant information and the tephritid name searches are currently undergoing revision. Other websites, such as the “True Fruit Flies of the Afrotropical Region” (De Meyer and White 2016) or the “PACIFLY” website, covering the Pacific region (Pest Management in the Pacific Project 2003), contain valuable information, but are limited in scope and are irregularly maintained due to sporadic funding. The checklist presented here is a global overview of valid species names of Dacini. By publishing this list in paper and digital format we hope to provide a resource for those studying fruit fly taxonomy as well as researchers concerned with their impacts on agriculture. The list is largely a consolidation of previous works, but following the results from recent phylogenetic work (Virgilio et al. 2015, San Jose et al. 2018 in press), we transfer one subgenus and eight species to different genera: Bactrocera subgenus Javadacus Hardy, considered to belong to the *Zeugodacus* group of subgenera by Hancock and Drew (2017), is transferred to genus *Zeugodacus*; *Bactrocera pseudocucurbitae* White, 1999, stat. rev., is transferred back to *Bactrocera* from *Zeugodacus*; *Zeugodacus arisanicus* Shiraki, 1933, stat. rev., is transferred back to *Zeugodacus* from *Bactrocera*; and *Z. brevipunctatus* (David & Hancock, 2017a), comb. n., *Z. javanensis* (Perkins, 1938), comb. n., *Z. montanus* (Hardy, 1983), comb. n., *Z. papuaensis* (Malloch, 1939), comb. n., *Z. scutellarius* (Bezzi, 1916), comb. n., *Z. semisurstyli* (Drew & Romig, 2013), comb. n., and *Z. trilineatus* (Hardy, 1955), comb. n. are transferred from *Bactrocera* to *Zeugodacus*.

## Methods

### Checklist

The source data is, for a large part, comprised of regional treatments (Drew 1989, Hancock and Drew 2006, White 2006, Drew and Romig 2013), with additions and revisions from more recent studies (Drew et al. 2011, Yu et al. 2012, Hancock 2015, Hancock and Drew 2015, Hendrichs et al. 2015, Schutze et al. 2015b, David et al. 2016, 2017, Drew and Hancock 2016, Freidberg et al. 2017, Han et al. 2017). Species included in the list are ordered alphabetically by genus. We do not indicate subgeneric or species complex ranks because their biological significance is, at present, unclear (Leblanc et al. 2015b). We do, however, provide the checklist also in spreadsheet form in supplementary material (S1) where these ranks are included and users can sort the species to their preference. For distribution, we use a coarse geographical indication: African or Asia-Pacific. The native region is indicated in the line with the species name, invasive regions are mentioned in the notes. We also include known male lure records for each species: cue-lure, methyl eugenol, isoeugenol and zingerone. Zingerone, first reported as a male lure by Tan and Nishida (2000), has received increased attention in recent years, with new lure records, including species not attracted to the two other lures, for a number of species in Australia and Papua New Guinea (Fay 2012, Royer et al. 2015, 2017). These records are included in the list, along with previously unpublished new records, indicated as such, from our team’s recent surveys carried out in Taiwan, Vietnam, Sri Lanka, Bangladesh and Nepal. For morphological terminology we follow White et al. (2000), which follows that in standard usage for other Diptera and differs somewhat from the older treatments.

### Conflicting views

For some species that have recently been synonymized or where there are conflicting views by different authors, we have indicated this under the ‘notes’ for the respective species, so that this may help users to place different views in perspective. It should also be noted that some authors do not follow the elevation of *Zeugodacus* to genus-level, because this is currently only supported by molecular data and morphological studies are inconclusive (David et al. 2017, Virgilio et al. 2015, San Jose et al. 2018). This affects the placement of nearly 200 species and although we agree that the re-assignment of species may have initially been premature, recent studies corroborate the need to recognize *Zeugodacus* as a genus to maintain *Bactrocera* as monophyletic. A 168-species seven-gene phylogeny, including multiple *Bactrocera* subgenera, shows that *Zeugodacus*, *Bactrocera* and *Dacus* each are monophyletic, and provides moderate statistical support for a sister relationship between *Zeugodacus* and *Dacus* (San Jose et al. 2018). A phylogeny with less representatives from Dacini, but 878 molecular loci, provides full statistical support for the sister relationship of *Zeugodacus* with *Dacus*, and *Bactrocera* as sister to both (Dupuis et al. 2017). As such, *Bactrocera* in the old sense is paraphyletic.

### Gender agreement

Because Dacini includes both masculine and feminine genera and because species have been moved between different genera over time, there is some confusion in the literature regarding the correct application of gender agreement. We have paid particular attention to this in the checklist. Most notably, several species names ending in -fer have originally been described without the author indicating if the name should be regarded as a noun or as an adjective. Following section 31.2.2 of the Zoological Code of Zoological Nomenclature (ICZN 1999), such names should be treated as a noun in apposition and the ending should not change when the species is moved to a different genus. This applies to *Bactrocera terminifer* (Walker, 1860), *B. speculifer* (Walker, 1865) and *B. curvifer* (Walker, 1860).

## Results

An overview of the current numbers of species split per genus, worldwide and according to the region where they are native, is shown in Table 1. Five species are shared between Africa and the Asia-Pacific regions: *Dacus ciliatus*, *Bactrocera oleae, B. dorsalis, B. latifrons* and *Zeugodacus cucurbitae*. There are no Dacini native to other regions, however, some species have become invasive in various countries outside their native distribution, such as *B. dorsalis, B. latifrons and Z. cucurbitae* from Asia introduced to Africa and various Pacific islands, *B. zonata* introduced from Asia to the Indian Ocean islands and the Middle East, including Egypt, and Asian *B. carambolae*, that invaded the Guianas and northern Brazil in South America. Two invasive species are native to Africa; *B. oleae* was introduced to the Mediterranean area, western Asia and California and northwestern Mexico in North America, and *D. ciliatus* has invaded the Middle East and the Indian subcontinent (Vargas et al. 2015).

**Table 1.** Number of described species per region.

	Worldwide	Africa	Asia-Pacific
Dacini	932	207	730
*Bactrocera*	461	13	451
*Zeugodacus*	196	1	195
*Dacus*	273	193	81
*Monacrostichus*	2	0	2

## Checklist

**Genus *Bactrocera* Macquart**

*Bactrocera abbreviata* (Hardy, 1974). Asia-Pacific. Non-pest. Zingerone.

Notes: Zingerone is a new lure record. *Bactrocera abbreviata* may be a junior synonym of *B. bipistulata*. There are morphological intermediates from Sri Lanka in the UHIM collection with a dark facial band and darker femora.

*Bactrocera abdofuscata* (Drew, 1971). Asia-Pacific. Non-pest.

*Bactrocera abdolonginqua* (Drew, 1971). Asia-Pacific. Non-pest. Methyl eugenol.

*Bactrocera abdomininigra* Drew, 1989. Asia-Pacific. Non-pest.

*Bactrocera abdonigella* (Drew, 1971). Asia-Pacific. Non-pest. Cue-lure.

*Bactrocera aberrans* (Hardy, 1951). Asia-Pacific. Non-pest. Isoeugenol.

Notes: Weakly attracted to isoeugenol (Royer 2015)

*Bactrocera abscondita* (Drew & Hancock, 1981). Asia-Pacific. Non-pest. Cue-lure.

*Bactrocera absidata* Drew, 1989. Asia-Pacific. Non-pest.

*Bactrocera abundans* Drew, 1989. Asia-Pacific. Non-pest. Cue-lure.

*Bactrocera aceraglans* White & Evenhuis, 1999. Asia-Pacific. Non-pest.

*Bactrocera aceromata* White & Evenhuis, 1999. Asia-Pacific. Non-pest.

*Bactrocera aemula* Drew, 1989 Asia-Pacific. Non-pest. Cue-lure.

*Bactrocera aenigmatica* (Malloch, 1931). Asia-Pacific. Non-pest.

*Bactrocera aeroginosa* (Drew & Hancock, 1981). Asia-Pacific. Non-pest. Cue-lure, zingerone.

*Bactrocera aethriobasis* (Hardy, 1973). Asia-Pacific. Non-pest. Methyl eugenol.

*Bactrocera affinibancroftii* Drew & Romig, 2013. Asia-Pacific. Non-pest. Methyl eugenol.

*Bactrocera affinidorsalis* (Hardy, 1982). Asia-Pacific. Non-pest. Cue-lure.

*Bactrocera affinis* (Hardy, 1954). Asia-Pacific. Non-pest. Methyl eugenol.

*Bactrocera aglaiae* (Hardy, 1951). Asia-Pacific. Non-pest. Methyl eugenol, zingerone.

*Bactrocera aithogaster* Drew, 1989. Asia-Pacific. Non-pest.

*Bactrocera albistrigata* de Meijere, 1911. Asia-Pacific. Fruit pest (polyphagous). Cue-lure.

Notes: *B. albistrigata* is very similar in appearance to *B. frauenfeldi*. Based on UHIM collection material, the morphological variation of both species is larger than Drew and Romig (2013) suggest. Hardy (1954) considered them synonyms, but they are treated as different species in Drew and Romig (2013). Their populations are likely allopatric, but there appears to be some morphological overlap.

*Bactrocera allwoodi* (Drew, 1979). Asia-Pacific. Non-pest. Cue-lure.

*Bactrocera alyxiae* (May, 1953). Asia-Pacific. Non-pest. Cue-lure, zingerone.

*Bactrocera amarambalensis* Drew, 2002. Asia-Pacific. Non-pest. Methyl eugenol.

*Bactrocera ampla* (Drew, 1971). Asia-Pacific. Non-pest. Cue-lure.

*Bactrocera amplexa* (Munro, 1984). Africa. Non-pest.

*Bactrocera amplexiseta* (May, 1962). Asia-Pacific. Non-pest. Methyl eugenol.

*Bactrocera andamanensis* (Kapoor, 1971). Asia-Pacific. Non-pest. Cue-lure.

*Bactrocera anfracta* Drew, 1989. Asia-Pacific. Non-pest. Cue-lure.

*Bactrocera angustifasciata* Drew, 1989. Asia-Pacific. Non-pest.

*Bactrocera anomala* (Drew, 1971). Asia-Pacific. Non-pest. Cue-lure.

*Bactrocera anthracina* (Drew, 1971). Asia-Pacific. Non-pest. Cue-lure.

*Bactrocera antigone* (Drew & Hancock, 1981). Asia-Pacific. Non-pest. Cue-lure.

*Bactrocera apicofuscans* White & Tsuruta, 2001. Asia-Pacific. Non-pest. Methyl eugenol.

*Bactrocera apiconigroscutella* Drew, 2002. Asia-Pacific. Non-pest. Cue-lure.

*Bactrocera apicopicta* Drew & Romig, 2013. Asia-Pacific. Non-pest. Cue-lure.

*Bactrocera aquila* (Drew, 1989). Asia-Pacific. Non-pest. Cue-lure.

*Bactrocera aquilonis* (May, 1965). Asia-Pacific. Fruit pest. Cue-lure.

Notes: May be a junior synonym of *B. tryoni*. The latter is supposedly restricted to the eastern side of Australia, *B. aquilonis* to the west. Even though these populations may now be largely allopatric, separated by the arid regions along the border between the Northern Territories and Queensland, they cannot be separated reliably based on morphology or using a population genetic approach with microsatellite data (Gilchrist et al. 2003, Cameron et al. 2010).

*Bactrocera arecae* (Hardy & Adachi, 1954). Asia-Pacific. Fruit pest (monophagous).

*Bactrocera assita* Drew, 1989 Asia-Pacific. Non-pest. Cue-lure.

*Bactrocera aterrima* (Drew, 1972). Asia-Pacific. Non-pest. Cue-lure.

*Bactrocera atra* (Malloch, 1938). Asia-Pacific. Non-pest. Cue-lure.

Notes: Cue-lure is a new lure record from surveys in French Polynesia in 1998.

*Bactrocera atrabifasciata* Drew & Romig, 2001. Asia-Pacific. Non-pest. Cue-lure.

*Bactrocera atramentata* (Hering, 1941). Asia-Pacific. Fruit pest (monophagous). Cue-lure, zingerone.

*Bactrocera atrifemur* Drew & Hancock, 1994. Asia-Pacific. Non-pest. Methyl eugenol.

*Bactrocera atriliniellata* Drew, 1989. Asia-Pacific. Non-pest. Cue-lure.

*Bactrocera aurantiaca* (Drew & Hancock, 1981). Asia-Pacific. Non-pest. Cue-lure.

*Bactrocera aurea* (May, 1952). Asia-Pacific. Non-pest. Zingerone.

*Bactrocera avittata* Drew & Romig, 2013. Asia-Pacific. Non-pest. Cue-lure.

*Bactrocera balagawii* Drew, 2011. Asia-Pacific. Non-pest. Methyl eugenol.

*Bactrocera bancroftii* (Tryon, 1927). Asia-Pacific. Fruit pest (oligophagous). Methyl eugenol.

*Bactrocera banneri* White, 1999. Asia-Pacific. Non-pest.

Notes: *B. banneri* and *B. coracina* are the two members of the subgenus Perkinsidacus in the most recent treatment of these species (Hancock and Drew 2017b), and both may belong in the genus *Zeugodacus*. They have the shallow emargination of sternite V and the long surstylus lobes of the male genitalia that fit with *Zeugodacus*, but lack a medial vitta on the scutum and the lateral vittae do not extend anteriorly beyond the transverse suture. Because there is, at present, no molecular data to support either placement and because it is unclear which, if any, of these morphological characters are apomorphic we tentatively leave both species in *Bactrocera*.

*Bactrocera barringtoniae* (Tryon, 1927). Asia-Pacific. Non-pest.

*Bactrocera batemani* Drew, 1989. Asia-Pacific. Non-pest. Methyl eugenol.

*Bactrocera beckerae* (Hardy, 1982). Asia-Pacific. Non-pest. Cue-lure.

*Bactrocera bellisi* Drew & Romig, 2013. Asia-Pacific. Non-pest. Cue-lure.

*Bactrocera bhutaniae* Drew & Romig, 2013. Asia-Pacific. Non-pest. Cue-lure.

*Bactrocera biarcuata* (Walker, 1865). Asia-Pacific. Non-pest. Methyl eugenol.

*Bactrocera bidentata* (May, 1963). Asia-Pacific. Non-pest.

*Bactrocera bifasciata* (Hardy, 1982). Asia-Pacific. Non-pest. Cue-lure.

*Bactrocera biguttula* (Bezzi, 1922). Africa. Non-pest.

*Bactrocera bimaculata* Drew & Hancock, 1994. Asia-Pacific. Non-pest. Cue-lure.

*Bactrocera binhduongiae* Drew & Romig, 2013. Asia-Pacific. Non-pest. Methyl eugenol.

*Bactrocera bipustulata* (Bezzi, 1914). Asia-Pacific. Non-pest. Cue-lure, zingerone.

Notes: Zingerone is a new lure record. See further comments under *B. abbreviata*.

*Bactrocera bitungiae* Drew & Romig, 2013. Asia-Pacific. Non-pest. Cue-lure.

*Bactrocera bivittata* Lin & Wang, 2005. Asia-Pacific. Non-pest. Methyl eugenol.

*Bactrocera blairiae* Drew & Romig, 2013. Asia-Pacific. Non-pest. Methyl eugenol.

*Bactrocera brachycera* (Bezzi, 1916). Asia-Pacific. Non-pest.

*Bactrocera breviaculeus* (Hardy, 1951). Asia-Pacific. Non-pest. Cue-lure, zingerone.

*Bactrocera brevistriata* (Drew, 1968). Asia-Pacific. Non-pest. Cue-lure.

*Bactrocera bruneiae* Drew & Romig, 2013. Asia-Pacific. Non-pest. Methyl eugenol.

*Bactrocera brunnea* (Perkins & May, 1949). Asia-Pacific. Non-pest.

*Bactrocera brunneola* White & Tsuruta, 2001. Asia-Pacific. Non-pest. Cue-lure.

*Bactrocera bryoniae* (Tryon, 1927). Asia-Pacific. Fruit pest (oligophagous). Cue-lure, zingerone.

*Bactrocera buinensis* Drew, 1989. Asia-Pacific. Non-pest. Cue-lure.

*Bactrocera bullata* Drew, 1989. Asia-Pacific. Non-pest.

*Bactrocera bullifera* (Hardy, 1973). Asia-Pacific. Non-pest.

*Bactrocera buloloensis* Drew, 1989. Asia-Pacific. Non-pest.

*Bactrocera cacuminata* (Hering, 1941). Asia-Pacific. Non-pest. Methyl eugenol.

*Bactrocera caledoniensis* Drew, 1989. Asia-Pacific. Non-pest. Cue-lure.

*Bactrocera caliginosa* (Hardy, 1970). Asia-Pacific. Non-pest.

*Bactrocera calophylli* (Perkins & May, 1949). Asia-Pacific. Non-pest.

*Bactrocera captiva* Drew & Romig, 2013. Asia-Pacific. Non-pest.

*Bactrocera carambolae* Drew & Hancock, 1994. Asia-Pacific. Fruit pest (polyphagous). Methyl eugenol, zingerone.

Notes: Under laboratory conditions, *B. carambolae* and *B. dorsalis* can produce fertile F1 hybrids, though with reduced survivability, and there is evidence for hybridization in the wild. Nonetheless, based on a combination of genetic and morphological evidence, they are considered to be two separate species (Ebina and Ohto 2006, Schutze et al. 2015a). The native distribution of *B. carambolae* is in Southeast Asia, but it is invasive in South America (Guianas and northern Brazil).

*Bactrocera carbonaria* (Hendel, 1927). Asia-Pacific. Non-pest. Cue-lure.

*Bactrocera careofascia* Drew & Romig, 2013. Asia-Pacific. Non-pest. Cue-lure.

*Bactrocera caryeae* (Kapoor, 1971). Asia-Pacific. Fruit pest (polyphagous). Methyl eugenol.

*Bactrocera ceylanica* Tsuruta & White, 2001. Asia-Pacific. Non-pest. Cue-lure.

*Bactrocera cheesmanae* (Perkins, 1939). Asia-Pacific. Non-pest. Methyl eugenol.

*Bactrocera chettalli* David & Ranganath, 2016. Asia-Pacific. Non-pest.

*Bactrocera cibodasae* Drew & Hancock, 1994. Asia-Pacific. Non-pest. Cue-lure.

*Bactrocera cinnabaria* Drew & Romig, 2013. Asia-Pacific. Non-pest.

*Bactrocera cinnamea* Drew, 1989. Asia-Pacific. Non-pest. Cue-lure.

*Bactrocera circamusae* Drew, 1989. Asia-Pacific. Non-pest. Cue-lure.

*Bactrocera citima* (Hardy, 1973). Asia-Pacific. Non-pest. Cue-lure.

*Bactrocera cogani* White, 2006. Africa. Non-pest.

*Bactrocera cognata* (Hardy & Adachi, 1954). Asia-Pacific. Non-pest.

*Bactrocera collita* Drew & Hancock, 1994. Asia-Pacific. Non-pest. Methyl eugenol.

*Bactrocera commensurata* Drew & Romig, 2013. Asia-Pacific. Non-pest. Methyl eugenol.

*Bactrocera commina* Drew, 1989. Asia-Pacific. Non-pest.

*Bactrocera confluens* (Drew, 1971). Asia-Pacific. Non-pest. Methyl eugenol.

*Bactrocera congener* Drew, 1989. Asia-Pacific. Non-pest. Cue-lure.

*Bactrocera consectorata* Drew, 1989. Asia-Pacific. Non-pest. Cue-lure.

*Bactrocera contermina* Drew, 1989. Asia-Pacific. Non-pest. Methyl eugenol.

*Bactrocera contigua* Drew, 1989. Asia-Pacific. Non-pest. Methyl eugenol.

*Bactrocera continua* (Bezzi, 1919). Asia-Pacific. Non-pest.

*Bactrocera coracina* (Drew, 1971). Asia-Pacific. Non-pest.

Notes: Maybe should be moved to *Zeugodacus*, see comments under *B. banneri*.

*Bactrocera correcta* (Bezzi, 1916). Asia-Pacific. Fruit pest. (polyphagous). Methyl eugenol.

*Bactrocera costalis* (Shiraki, 1933). Asia-Pacific. Non-pest. Cue-lure.

*Bactrocera curreyi* Drew, 1989. Asia-Pacific. Non-pest. Cue-lure.

*Bactrocera curtivitta* Drew & Romig, 2013. Asia-Pacific. Non-pest.

*Bactrocera curvifer* (Walker, 1864). Asia-Pacific. Non-pest. Methyl eugenol.

*Bactrocera curvipennis* (Froggatt, 1909). Asia-Pacific. Fruit pest. Cue-lure.

*Bactrocera curvosterna* Drew & Romig, 2013. Asia-Pacific. Non-pest. Cue-lure.

*Bactrocera dapsiles* Drew, 1989. Asia-Pacific. Non-pest. Methyl eugenol.

*Bactrocera daruensis* Drew, 1989. Asia-Pacific. Non-pest.

*Bactrocera decumana* (Drew, 1972). Asia-Pacific. Non-pest. Cue-lure.

*Bactrocera decurtans* (May, 1965). Asia-Pacific. Non-pest. Methyl eugenol.

*Bactrocera diallagma* Drew, 1989. Asia-Pacific. Non-pest. Methyl eugenol.

*Bactrocera diaphana* (Hering, 1953). Asia-Pacific. Non-pest.

*Bactrocera digressa* Radhakrishnan, 1999. Asia-Pacific. Non-pest. Cue-lure, zingerone.

Notes: Zingerone is a new lure record.

*Bactrocera diospyri* Drew, 1989. Asia-Pacific. Non-pest.

*Bactrocera dispar* (Hardy, 1982). Asia-Pacific. Non-pest.

*Bactrocera distincta* (Malloch, 1931). Asia-Pacific. Fruit pest. Cue-lure.

*Bactrocera dongnaiae* Drew & Romig, 2013. Asia-Pacific. Non-pest. Cue-lure.

*Bactrocera dorsalis* (Hendel, 1912). Asia-Pacific. Fruit pest (polyphagous). Methyl eugenol, zingerone.

Notes: *B. dorsalis*, the Oriental fruit fly, is one of the most significant pest species within the Tephritidae, and it is invasive in many areas of Asia, Africa and the Pacific islands (Vargas et al. 2015). Based on a total-evidence approach, *B. papayae*, *B. invadens* and *B. philippinensis* are now considered synonyms of *B. dorsalis*, but these names can still be found in numerous papers and internet website resources. *Bactrocera dorsalis* is known to hybridize with *B. carambolae* and genetic evidence suggests that there is historic hybridization with *B. kandiensis* (Schutze et al. 2015b); see notes under those respective species for further details.

*Bactrocera dorsaloides* (Hardy & Adachi, 1954). Asia-Pacific. Non-pest.

*Bactrocera dyscrita* (Drew, 1971). Asia-Pacific. Non-pest. Cue-lure.

*Bactrocera ebenea* (Drew, 1971). Asia-Pacific. Non-pest. Methyl eugenol.

*Bactrocera ektoalangiae* Drew & Hancock, 1999. Asia-Pacific. Non-pest.

*Bactrocera elongata* Drew & Romig, 2013. Asia-Pacific. Non-pest. Cue-lure.

*Bactrocera endiandrae* (Perkins & May, 1949). Asia-Pacific. Non-pest. Methyl eugenol.

*Bactrocera enochra* (Drew, 1972). Asia-Pacific. Non-pest. Cue-lure.

*Bactrocera epicharis* (Hardy, 1970). Asia-Pacific. Non-pest. Cue-lure.

*Bactrocera erubescentis* (Drew & Hancock, 1981). Asia-Pacific. Non-pest. Cue-lure.

*Bactrocera eurycosta* Drew & Romig, 2013. Asia-Pacific. Non-pest. Cue-lure.

*Bactrocera exigua* (May, 1958). Asia-Pacific. Non-pest.

*Bactrocera eximia* Drew, 1989. Asia-Pacific. Non-pest.

*Bactrocera expandens* (Walker, 1859). Asia-Pacific. Fruit pest.

*Bactrocera exspoliata* (Hering, 1941). Asia-Pacific. Non-pest.

*Bactrocera facialis* (Coquillett, 1909). Asia-Pacific. Fruit pest. Cue-lure.

*Bactrocera fagraea* (Tryon, 1927). Asia-Pacific. Non-pest. Cue-lure.

*Bactrocera fastigata* Tsuruta & White, 2001. Asia-Pacific. Non-pest. Cue-lure.

*Bactrocera fergussoniensis* Drew, 1989. Asia-Pacific. Non-pest.

*Bactrocera fernandoi* Tsuruta & White, 2001. Asia-Pacific. Non-pest. Cue-lure.

*Bactrocera finitima* Drew, 1989. Asia-Pacific. Non-pest.

*Bactrocera flavinotus* (May, 1957). Asia-Pacific. Non-pest.

*Bactrocera flavipennis* (Hardy 1982). Asia-Pacific. Non-pest. Cue-lure.

*Bactrocera flavoscutellata* Lin & Wang, 2005. Asia-Pacific. Non-pest. Cue-lure.

Notes: This is likely a junior synonym of *B. pernigra*. The only distinguishing character is in the width of the basal dark band on the scutellum, but this appears to be variable (Drew and Romig 2013). Because the characters have only been studied in small sample sizes there has not yet been an official synonymy.

*Bactrocera flavosterna* Drew & Romig, 2013. Asia-Pacific. Non-pest. Cue-lure.

*Bactrocera floresiae* Drew & Hancock, 1994. Asia-Pacific. Non-pest. Methyl eugenol.

*Bactrocera frauenfeldi* (Schiner, 1868). Asia-Pacific. Fruit pest (polyphagous). Cue-lure, zingerone.

Notes: See under *B. albistrigata*.

*Bactrocera froggatti* (Bezzi, 1928). Asia-Pacific. Non-pest. Methyl eugenol.

*Bactrocera fuliginus* (Drew & Hancock, 1981). Asia-Pacific. Non-pest. Cue-lure.

*Bactrocera fulvicauda* (Perkins, 1939). Asia-Pacific. Non-pest. Methyl eugenol.

*Bactrocera fulvifacies* (Perkins, 1939). Asia-Pacific. Non-pest.

*Bactrocera fulvifemur* Drew & Hancock, 1994. Asia-Pacific. Non-pest. Cue-lure.

*Bactrocera fulvosterna* Drew & Romig, 2013. Asia-Pacific. Non-pest. Cue-lure.

*Bactrocera furcata* David & Hancock, 2017. Asia-Pacific. Non-pest.

*Bactrocera furfurosa* Drew, 1989. Asia-Pacific. Non-pest. Cue-lure.

*Bactrocera furvescens* Drew, 1989. Asia-Pacific. Non-pest. Cue-lure.

*Bactrocera furvilineata* Drew, 1989. Asia-Pacific. Non-pest. Cue-lure.

*Bactrocera fuscalata* Drew, 1989. Asia-Pacific. Non-pest. Methyl eugenol.

*Bactrocera fuscitibia* Drew & Hancock, 1994. Asia-Pacific. Non-pest. Cue-lure.

*Bactrocera fuscoformosa* Drew & Romig, 2013. Asia-Pacific. Non-pest. Cue-lure.

*Bactrocera fuscohumeralis* White & Evenhuis, 1999. Asia-Pacific. Non-pest.

*Bactrocera fuscolobata* Drew & Romig, 2013. Asia-Pacific. Non-pest. Cue-lure.

*Bactrocera fuscoptera* Drew & Romig, 2013. Asia-Pacific. Non-pest. Methyl eugenol.

*Bactrocera garciniae* Bezzi, 1913. Asia-Pacific. Non-pest.

*Bactrocera gnetum* Drew & Hancock, 1995. Asia-Pacific. Non-pest.

*Bactrocera gombokensis* Drew & Hancock, 1994. Asia-Pacific. Non-pest. Cue-lure.

*Bactrocera grandifasciata* White & Evenhuis, 1999. Asia-Pacific. Non-pest.

*Bactrocera grandistylus* Drew & Hancock, 1995. Asia-Pacific. Non-pest.

*Bactrocera halfordiae* (Tryon, 1927). Asia-Pacific. Fruit pest.

*Bactrocera halmaherae* Drew & Romig, 2013. Asia-Pacific. Non-pest. Cue-lure.

*Bactrocera hantanae* Tsuruta & White, 2001. Asia-Pacific. Non-pest. Cue-lure.

*Bactrocera harrietensis* Ramani & David, 2016. Asia-Pacific. Non-pest.

*Bactrocera hastigerina* (Hardy, 1954). Asia-Pacific. Fruit pest (monophagous).

*Bactrocera hispidula* (May, 1958). Asia-Pacific. Non-pest.

*Bactrocera hollingsworthi* Drew & Romig, 2001. Asia-Pacific. Non-pest. Cue-lure.

*Bactrocera holtmanni* (Hardy, 1974). Asia-Pacific. Non-pest. Cue-lure.

*Bactrocera humilis* (Drew & Hancock, 1981). Asia-Pacific. Non-pest.

*Bactrocera hyalina* (Shiraki, 1933). Asia-Pacific. Non-pest.

*Bactrocera hypomelaina* Drew, 1989. Asia-Pacific. Non-pest. Cue-lure.

*Bactrocera icelus* (Hardy, 1974). Asia-Pacific. Non-pest.

Notes: We continue the use of a masculine epithet like in previous treatments. Hardy did not give an etymology in his description of the species, but ‘icelus’ could refer to the Greek mythical figure by that name, or reference to the Greek word for ‘appearance’, and we treat it as a noun in aposition.

*Bactrocera illusioscutellaris* Drew & Romig, 2013. Asia-Pacific. Non-pest. Cue-lure, zingerone.

Notes: Zingerone is a new lure record.

*Bactrocera impunctata* (de Mejeire, 1914). Asia-Pacific. Non-pest. Methyl eugenol.

*Bactrocera incompta* Drew & Romig, 2013. Asia-Pacific. Non-pest. Cue-lure.

*Bactrocera inconspicua* Drew & Romig, 2013. Asia-Pacific. Non-pest. Methyl eugenol.

*Bactrocera inconstans* Drew, 1989. Asia-Pacific. Non-pest. Cue-lure.

*Bactrocera indecora* (Drew 1971). Asia-Pacific. Non-pest. Cue-lure.

*Bactrocera indonesiae* Drew & Hancock, 1994. Asia-Pacific. Non-pest. Methyl eugenol. Zingerone.

*Bactrocera infulata* Drew & Hancock, 1994. Asia-Pacific. Non-pest. Methyl eugenol.

*Bactrocera invisitata* Drew, 1989. Asia-Pacific. Non-pest. Methyl eugenol.

*Bactrocera involuta* (Hardy, 1982). Asia-Pacific. Non-pest. Cue-lure.

*Bactrocera irvingiae* Drew & Hancock, 1994. Asia-Pacific. Non-pest.

*Bactrocera ismayi* Drew, 1989. Asia-Pacific. Non-pest. Methyl eugenol.

Notes: Methyl eugenol is a new lure record from surveys in Papua New Guinea in 1997/1999.

*Bactrocera jaceobancroftii* Drew & Romig, 2013. Asia-Pacific. Non-pest. Methyl eugenol.

*Bactrocera jarvisi* (Tryon, 1927). Asia-Pacific. Fruit pest. Cue-lure, zingerone.

*Bactrocera kalimantaniae* Drew & Romig, 2013. Asia-Pacific. Non-pest. Cue-lure.

*Bactrocera kanchanaburi* Drew & Hancock ,1994. Asia-Pacific. Non-pest. Methyl eugenol.

*Bactrocera kandiensis* Drew & Hancock, 1994. Asia-Pacific. Fruit pest (polyphagous). Methyl eugenol.

Notes: There is likely some (historical) introgression or hybridization between *B. kandiensis* and *B. dorsalis*, and the two cannot be separated reliably using mitochondrial genes (Schutze et al. 2015a, 2015b, San Jose, unpublished data).

*Bactrocera kelaena* Drew, 1989. Asia-Pacific. Non-pest. Methyl eugenol.

*Bactrocera kinabalu* Drew & Hancock, 1994. Asia-Pacific. Non-pest. Cue-lure.

*Bactrocera kirki* (Froggatt, 1910). Asia-Pacific. Fruit pest. Cue-lure.

*Bactrocera kohkongiae* Leblanc, 2015. Asia-Pacific. Non-pest. Cue-lure.

*Bactrocera kraussi* (Hardy, 1951). Asia-Pacific. Fruit pest. Cue-lure.

*Bactrocera kuniyoshii* (Shiraki, 1968). Asia-Pacific. Non-pest.

*Bactrocera laithieuiae* Drew & Romig, 2013. Asia-Pacific. Non-pest. Cue-lure.

*Bactrocera lampabilis* (Drew, 1971). Asia-Pacific. Non-pest. Methyl eugenol.

*Bactrocera lata* (Perkins 1938). Asia-Pacific. Non-pest. Cue-lure.

*Bactrocera lateritaenia* Drew & Hancock, 1994. Asia-Pacific. Non-pest. Cue-lure.

*Bactrocera laticaudus* (Hardy, 1950). Asia-Pacific. Non-pest. Methyl eugenol.

*Bactrocera laticosta* Drew, 1989. Asia-Pacific. Non-pest. Cue-lure.

*Bactrocera latifrons* (Hendel, 1915). Asia-Pacific. Fruit pest (oligophagous).

Notes: Native to Asia and introduced into Africa and Hawaii.

*Bactrocera latilineata* Drew, 1989. Asia-Pacific. Non-pest.

Notes: Male attractant uncertain, previous lure records are likely incorrect (see Drew 1989).

*Bactrocera latilineola* Drew & Hancock, 1994. Asia-Pacific. Non-pest. Methyl eugenol.

*Bactrocera latissima* Drew, 1989. Asia-Pacific. Non-pest. Cue-lure.

*Bactrocera limbifera* (Bezzi, 1919). Asia-Pacific. Non-pest. Cue-lure.

*Bactrocera linduensis* Drew & Romig, 2013. Asia-Pacific. Non-pest. Cue-lure.

*Bactrocera lineata* (Perkins, 1939). Asia-Pacific. Fruit pest (monophagous). Cue-lure.

*Bactrocera lombokensis* Drew & Hancock, 1994. Asia-Pacific. Non-pest. Cue-lure.

*Bactrocera longicornis* Macquart, 1835. Asia-Pacific. Non-pest. Cue-lure.

Notes: Type species for the genus (see Hardy 1976).

*Bactrocera lucida* (Munro, 1939). Africa. Non-pest.

*Bactrocera luteola* (Malloch, 1931). Asia-Pacific. Non-pest.

*Bactrocera maculigera* Doleschall, 1858. Asia-Pacific. Non-pest. Methyl eugenol.

*Bactrocera makilingensis* Drew & Hancock, 1994. Asia-Pacific. Non-pest. Cue-lure.

*Bactrocera malaysiensis* Drew & Hancock, 1994. Asia-Pacific. Non-pest. Cue-lure.

*Bactrocera mamaliae* Drew & Romig, 2013. Asia-Pacific. Non-pest. Cue-lure.

*Bactrocera manskii* (Perkins & May, 1949). Asia-Pacific. Non-pest. Cue-lure.

*Bactrocera matsumurai* (Shiraki, 1933). Asia-Pacific. Non-pest.

*Bactrocera mayi* (Hardy, 1951). Asia-Pacific. Non-pest. Methyl eugenol.

*Bactrocera mcgregori* (Bezzi, 1919). Asia-Pacific. Non-pest.

*Bactrocera mediorufula* Drew & Romig, 2013. Asia-Pacific. Non-pest. Methyl eugenol.

*Bactrocera megaspilus* (Hardy, 1982). Asia-Pacific. Non-pest. Cue-lure.

*Bactrocera melania* (Hardy & Adachi, 1954). Asia-Pacific. Non-pest.

*Bactrocera melanogaster* Drew, 1989. Asia-Pacific. Non-pest. Methyl eugenol.

*Bactrocera melanoscutata* Drew, 1989. Asia-Pacific. Non-pest.

*Bactrocera melanothoracica* Drew, 1989. Asia-Pacific. Non-pest. Methyl eugenol.

*Bactrocera melanotus* (Coquillett, 1909). Asia-Pacific. Fruit pest. Cue-lure.

*Bactrocera melas* (Perkins & May, 1949). Asia-Pacific. Fruit pest. Cue-lure.

Notes: It is uncertain if *B. melas* is a distinct species. Specimens identified as *B. melas* may be a dark form of *B. tryoni*, or hybrids of *B. tryoni* and *B. neohumeralis* (see Hancock et al. 2000).

*Bactrocera melastomatos* Drew & Hancock, 1994. Asia-Pacific. Non-pest. Cue-lure.

*Bactrocera memnonia* (Drew, 1989). Asia-Pacific. Non-pest. Methyl eugenol.

*Bactrocera menanus* (Munro, 1984). Africa. Non-pest.

*Bactrocera mendosa* (May, 1958). Asia-Pacific. Non-pest.

*Bactrocera merapiensis* Drew & Hancock, 1994. Asia-Pacific. Non-pest. Cue-lure.

*Bactrocera mesomelas* (Bezzi, 1908a). Africa. Fruit pest (monophagous).

*Bactrocera mesonotaitha* Drew, 1989. Asia-Pacific. Non-pest.

*Bactrocera mesonotochra* Drew, 1989. Asia-Pacific. Non-pest.

*Bactrocera mimulus* Drew, 1989. Asia-Pacific. Non-pest. Methyl eugenol.

*Bactrocera minax* (Enderlein, 1920). Asia-Pacific. Fruit pest.

*Bactrocera minuscula* Drew & Hancock, 1994. Asia-Pacific. Non-pest. Methyl eugenol.

*Bactrocera minuta* (Drew, 1971). Asia-Pacific. Non-pest. Cue-lure.

*Bactrocera moluccensis* (Perkins, 1939). Asia-Pacific. Fruit pest (monophagous). Cue-lure, zingerone.

*Bactrocera montyanus* (Munro, 1984). Africa. Non-pest.

*Bactrocera morobiensis* Drew, 1989. Asia-Pacific. Non-pest. Cue-lure.

*Bactrocera morula* Drew, 1989. Asia-Pacific. Non-pest. Cue-lure.

*Bactrocera mucronis* (Drew, 1971). Asia-Pacific. Fruit pest. Cue-lure.

*Bactrocera muiri* (Hardy & Adachi, 1954). Asia-Pacific. Non-pest.

*Bactrocera munroi* White, 2004. Africa. Non-pest.

*Bactrocera murrayi* (Perkins, 1939). Asia-Pacific. Fruit pest. Zingerone.

*Bactrocera musae* (Tryon, 1927). Asia-Pacific. Fruit pest (oligophagous). Methyl eugenol.

*Bactrocera mutabilis* (May, 1952). Asia-Pacific. Fruit pest.

*Bactrocera nanoarcuata* Drew & Romig, 2013. Asia-Pacific. Non-pest. Cue-lure.

*Bactrocera nationigrotibialis* Drew & Romig, 2013. Asia-Pacific. Non-pest. Methyl eugenol.

*Bactrocera naucleae* Drew & Romig, 2001. Asia-Pacific. Non-pest. Methyl eugenol.

*Bactrocera neoarecae* Drew, 2002. Asia-Pacific. Non-pest. Methyl eugenol.

*Bactrocera neocheesmanae* Drew, 1989. Asia-Pacific. Non-pest. Methyl eugenol.

*Bactrocera neocognata* Drew & Hancock, 1994. Asia-Pacific. Non-pest. Cue-lure.

*Bactrocera neofulvicauda* Drew & Romig, 2013. Asia-Pacific. Non-pest. Cue-lure.

*Bactrocera neohumeralis* (Hardy, 1951). Asia-Pacific. Fruit pest. Cue-lure, zingerone.

*Bactrocera neonigrita* Drew, 1989. Asia-Pacific. Non-pest. Methyl eugenol.

*Bactrocera neonigrotibialis* Drew, 2002. Asia-Pacific. Non-pest. Cue-lure.

*Bactrocera neopagdeni* Drew, 1989. Asia-Pacific. Non-pest.

*Bactrocera neopropinqua* Drew & Hancock, 1994. Asia-Pacific. Non-pest.

*Bactrocera neoritsemai* Drew & Romig, 2013. Asia-Pacific. Non-pest. Cue-lure.

*Bactrocera neoxanthodes* Drew & Romig, 2001. Asia-Pacific. Non-pest.

*Bactrocera nesiotes* (Munro, 1984). Africa. Non-pest.

*Bactrocera nigella* (Drew, 1968). Asia-Pacific. Non-pest. Methyl eugenol.

*Bactrocera nigra* (Tryon, 1927). Asia-Pacific. Non-pest.

*Bactrocera nigrescens* (Drew, 1968). Asia-Pacific. Non-pest. Methyl eugenol.

*Bactrocera nigrescentis* (Drew, 1971). Asia-Pacific. Non-pest. Cue-lure.

*Bactrocera nigricula* (Drew, 1989). Asia-Pacific. Non-pest.

*Bactrocera nigrifacia* Zhang Ji & Chen, 2011. Asia-Pacific. Non-pest. Cue-lure.

*Bactrocera nigrifemorata* Li & Wang, 2011. Asia-Pacific. Non-pest.

*Bactrocera nigrita* (Hardy, 1955). Asia-Pacific. Non-pest. Methyl eugenol.

*Bactrocera nigrivenata* (Munro, 1937). Africa. Non-pest.

*Bactrocera nigrofemoralis* White & Tsuruta, 2001. Asia-Pacific. Non-pest. Cue-lure.

*Bactrocera nigroscutata* White & Evenhuis, 1999. Asia-Pacific. Non-pest.

*Bactrocera nigrotibialis* (Perkins, 1938). Asia-Pacific. Fruit pest (oligophagous). Cue-lure.

*Bactrocera nigrovittata* Drew, 1989. Asia-Pacific. Non-pest.

*Bactrocera notatagena* (May, 1953). Asia-Pacific. Non-pest.

*Bactrocera nothaphoebe* Drew & Romig, 2013. Asia-Pacific. Non-pest.

*Bactrocera obfuscata* Drew, 1989. Asia-Pacific. Non-pest. Cue-lure.

*Bactrocera oblineata* Drew, 1989. Asia-Pacific. Non-pest. Cue-lure.

*Bactrocera obliqua* (Malloch, 1939). Asia-Pacific. Fruit pest.

*Bactrocera obliquivenosa* Drew & Romig, 2001. Asia-Pacific. Non-pest. Methyl eugenol.

*Bactrocera obscura* (Malloch, 1931). Asia-Pacific. Non-pest. Cue-lure.

*Bactrocera obscurata* (de Mejeire, 1911). Asia-Pacific. Non-pest.

*Bactrocera obscurivitta* Drew & Romig, 2013. Asia-Pacific. Non-pest. Cue-lure.

*Bactrocera obtrullata* White & Evenhuis, 1999. Asia-Pacific. Non-pest.

*Bactrocera occipitalis* (Bezzi, 1919). Asia-Pacific. Fruit pest. Methyl eugenol.

Notes: The pest status of this species is uncertain and has possibly been overrated in literature, based on a few obscure rearing records cited in Drew and Hancock (1994).

*Bactrocera ochracea* Drew, 1989. Asia-Pacific. Non-pest. Cue-lure.

*Bactrocera ochroma* Drew & Romig, 2013. Asia-Pacific. Fruit pest (monophagous). Methyl eugenol.

*Bactrocera ochromarginis* (Drew, 1971). Asia-Pacific. Non-pest. Methyl eugenol.

*Bactrocera ochrosiae* (Malloch, 1942). Asia-Pacific. Non-pest. Cue-lure.

*Bactrocera ochroventer* Drew & Romig, 2013. Asia-Pacific. Non-pest.

Notes: Male attractant uncertain. Label data of collected specimens suggests that they have been collected both with cue lure and methyl eugenol, which seems unlikely. Possibly the traps have been contaminated.

*Bactrocera oleae* (Gmelin, 1790). Africa. Fruit pest (monophagous).

Notes: *Bactrocera oleae* is thought to be native to sub-Saharan Africa, and invasive in North Africa, southern Europe, western Asia, and California and northwestern Mexico in North America.

*Bactrocera opacovitta* Drew & Romig, 2013. Asia-Pacific. Non-pest. Methyl eugenol.

*Bactrocera opiliae* (Drew & Hardy, 1981). Asia-Pacific. Non-pest. Methyl eugenol.

*Bactrocera osbeckiae* Drew & Hancock, 1994. Asia-Pacific. Non-pest.

*Bactrocera pacificae* Drew & Romig, 2001. Asia-Pacific. Non-pest.

*Bactrocera pagdeni* (Malloch, 1939). Asia-Pacific. Non-pest.

*Bactrocera pallescentis* (Hardy, 1955). Asia-Pacific. Non-pest.

*Bactrocera pallida* (Perkins & May, 1949). Asia-Pacific. Non-pest. Methyl eugenol.

*Bactrocera paraarecae* Drew & Romig, 2013. Asia-Pacific. Non-pest. Methyl eugenol.

*Bactrocera parabancroftii* Drew, 2011. Asia-Pacific. Non-pest. Cue-lure.

*Bactrocera parabarringtoniae* Drew & Hancock, 1999. Asia-Pacific. Non-pest. Cue-lure.

*Bactrocera paradiospyri* Chen Zhou & Li, 2011. Asia-Pacific. Non-pest. Methyl eugenol.

*Bactrocera parafrauenfeldi* Drew, 1989. Asia-Pacific. Non-pest. Cue-lure.

*Bactrocera parafroggatti* Drew & Romig, 2001. Asia-Pacific. Non-pest. Methyl eugenol.

*Bactrocera paralatissima* Drew & Romig, 2013. Asia-Pacific. Non-pest. Cue-lure.

*Bactrocera paralimbifera* Drew & Romig, 2013. Asia-Pacific. Non-pest. Cue-lure.

*Bactrocera paramusae* Drew, 1989. Asia-Pacific. Non-pest. Cue-lure.

*Bactrocera paranigrita* Drew & Romi,g 2013. Asia-Pacific. Non-pest. Methyl eugenol.

*Bactrocera paraosbeckiae* Drew, 2002. Asia-Pacific. Non-pest. Cue-lure.

*Bactrocera paraverbascifoliae* Drew, 2002. Asia-Pacific. Non-pest. Methyl eugenol.

*Bactrocera paraxanthodes* Drew & Hancock, 1995. Asia-Pacific. Non-pest. Methyl eugenol.

Notes: The attraction to methyl eugenol possibly is weak.

*Bactrocera parvula* (Hendel, 1912). Asia-Pacific. Non-pest.

*Bactrocera passiflorae* (Froggatt, 1910). Asia-Pacific. Fruit pest. Cue-lure.

*Bactrocera patula* Drew & Romig, 2013. Asia-Pacific. Non-pest. Cue-lure.

*Bactrocera pectoralis* (Walker, 1859). Asia-Pacific. Non-pest.

*Bactrocera pedestris* (Bezzi, 1913). Asia-Pacific. Non-pest. Cue-lure.

*Bactrocera pendleburyi* (Perkins, 1938). Asia-Pacific. Non-pest. Zingerone.

Notes: Zingerone is a new lure record.

*Bactrocera peneallwoodi* Drew & Romig, 2013. Asia-Pacific. Non-pest.

Notes: Male attractant uncertain. Label data of collected specimens suggests that they have been collected both with cue lure and methyl eugenol, which seems unlikely. Possibly the traps have been contaminated.

*Bactrocera penebeckerae* Drew & Romig, 2013. Asia-Pacific. Non-pest.

*Bactrocera penecognata* Drew & Hancock, 1994. Asia-Pacific. Non-pest. Cue-lure.

*Bactrocera penecorrecta* Drew, 2002. Asia-Pacific. Non-pest. Methyl eugenol.

*Bactrocera penecostalis* Drew & Romig, 2013. Asia-Pacific. Non-pest. Cue-lure.

*Bactrocera penefurva* Drew, 1989. Asia-Pacific. Non-pest.

*Bactrocera peneobscura* Drew & Romig, 2001. Asia-Pacific. Non-pest. Cue-lure.

*Bactrocera penephaea* Drew & Romig, 2013. Asia-Pacific. Non-pest. Cue-lure.

*Bactrocera peninsularis* (Drew & Hancock, 1981). Asia-Pacific. Non-pest. Cue-lure.

*Bactrocera pepisalae* (Froggatt, 1910). Asia-Pacific. Non-pest. Methyl eugenol.

*Bactrocera perfusca* (Aubertin, 1929). Asia-Pacific. Fruit pest.

*Bactrocera perigrapha* White & Tsuruta, 2001. Asia-Pacific. Non-pest. Cue-lure, zingerone.

Notes: Zingerone is a new lure record.

*Bactrocera perkinsi* (Drew & Hancock, 1981). Asia-Pacific. Non-pest. Cue-lure.

*Bactrocera pernigra* Ito, 1983. Asia-Pacific. Non-pest. Cue-lure.

Notes: see comments under *B. flavoscutellata*

*Bactrocera peterseni* (Hardy, 1970). Asia-Pacific. Non-pest.

*Bactrocera petila* Drew, 1989. Asia-Pacific. Non-pest. Cue-lure.

*Bactrocera phaea* (Drew, 1971). Asia-Pacific. Non-pest. Cue-lure.

*Bactrocera phaleriae* (May, 1956). Asia-Pacific. Non-pest.

*Bactrocera picea* (Drew, 1972). Asia-Pacific. Non-pest. Methyl eugenol.

*Bactrocera pictipennis* Lin & Zeng, 2011. Asia-Pacific. Non-pest. Methyl eugenol.

*Bactrocera pisinna* Drew, 1989. Asia-Pacific. Non-pest. Cue-lure.

*Bactrocera popondettiensis* Drew, 1989. Asia-Pacific. Non-pest.

*Bactrocera profunda* Tsuruta & White, 2001. Asia-Pacific. Non-pest. Cue-lure.

*Bactrocera prolixa* Drew, 1989. Asia-Pacific. Non-pest. Methyl eugenol.

*Bactrocera propedistincta* Drew, 1989. Asia-Pacific. Non-pest.

*Bactrocera propinqua* (Hardy & Adachi, 1954). Asia-Pacific. Non-pest. Cue-lure.

*Bactrocera pruniae* Drew & Romig, 2013. Asia-Pacific. Fruit pest (monophagous).

*Bactrocera pseudobeckerae* Drew & Romig, 2013. Asia-Pacific. Non-pest. Cue-lure.

*Bactrocera pseudocucurbitae* White, 1999, stat. rev. Asia-Pacific. Non-pest. Cue-lure.

Notes: This species was assigned to the subgenus Parasinodacus by Drew and Romig (2013), and subsequently assigned to genus *Zeugodacus* by De Meyer et al. (2015). It was assigned to *Parasinodacus* based on having a medial yellow scutal vitta and having just two scutellar setae, but it differs from other members of *Parasinodacus* in lacking yellow marks anterior to the transverse suture (= notopleural suture of Drew and Romig 2013), the presence of which is likely a reliable character for assignment to *Zeugodacus* (White 1999, San Jose et al. 2018). In a phylogeny based on molecular data from seven genes, the species is reliably placed within the *Bactrocera* clade (San Jose et al. 2018). We therefore here move the species back to *Bactrocera* and tentatively assign it to the subgenus Bactrocera.

*Bactrocera pseudodistincta* (Drew, 1971). Asia-Pacific. Non-pest. Cue-lure.

*Bactrocera pseudoversicolor* Drew, 2002. Asia-Pacific. Non-pest. Methyl eugenol.

*Bactrocera psidii* (Froggatt, 1899). Asia-Pacific. Fruit pest. Cue-lure.

*Bactrocera pulchra* Tryon, 1927. Asia-Pacific. Non-pest.

*Bactrocera pusilla* (Hardy, 1983). Asia-Pacific. Non-pest. Cue-lure.

*Bactrocera pyrifoliae* Drew & Hancock, 1994. Asia-Pacific. Fruit pest (oligophagous).

*Bactrocera quadrata* (May, 1963). Asia-Pacific. Non-pest. Cue-lure.

*Bactrocera quadrisetosa* (Bezzi, 1928). Asia-Pacific. Fruit pest.

*Bactrocera quasiinfulata* Drew & Romig, 2013. Asia-Pacific. Non-pest. Cue-lure.

*Bactrocera quasineonigrita* Drew & Romig, 2013. Asia-Pacific. Non-pest. Methyl eugenol.

*Bactrocera quasipropinqua* Drew & Hancock, 1994. Asia-Pacific. Non-pest.

*Bactrocera quasisilvicola* Drew, 1989. Asia-Pacific. Non-pest. Cue-lure.

*Bactrocera raiensis* Drew & Hancock, 1994. Asia-Pacific. Non-pest. Methyl eugenol.

*Bactrocera ramuensis* Drew, 2011. Asia-Pacific. Non-pest. Cue-lure.

*Bactrocera ranganathi* Drew & Romig, 2013. Asia-Pacific. Non-pest. Methyl eugenol.

*Bactrocera reclinata* Drew, 1989. Asia-Pacific. Non-pest. Methyl eugenol.

*Bactrocera recurrens* (Hering, 1941). Asia-Pacific. Non-pest. Cue-lure.

*Bactrocera redunca* (Drew, 1971). Asia-Pacific. Non-pest. Cue-lure.

*Bactrocera repanda* Drew, 1989. Asia-Pacific. Non-pest.

*Bactrocera resima* (Drew, 1971). Asia-Pacific. Non-pest. Cue-lure.

*Bactrocera retrorsa* Drew, 1989. Asia-Pacific. Non-pest. Methyl eugenol.

*Bactrocera rhabdota* Drew, 1989. Asia-Pacific. Non-pest. Cue-lure.

*Bactrocera ritsemai* (Weyenbergh, 1869). Asia-Pacific. Non-pest. Cue-lure.

*Bactrocera robertsi* Drew, 1989. Asia-Pacific. Non-pest. Cue-lure.

*Bactrocera robiginosa* (May, 1958). Asia-Pacific. Non-pest.

*Bactrocera romigae* (Drew & Hancock, 1981). Asia-Pacific. Non-pest. Methyl eugenol.

*Bactrocera rubigina* (Wang & Zhao, 1989). Asia-Pacific. Non-pest. Cue-lure, zingerone.

Notes: Zingerone is a new lure record.

*Bactrocera rufescens* (May, 1967). Asia-Pacific. Non-pest. Cue-lure.

*Bactrocera rufivitta* Drew, 2011. Asia-Pacific. Non-pest. Cue-lure.

*Bactrocera rufofuscula* (Drew & Hancock, 1981). Asia-Pacific. Non-pest. Cue-lure, zingerone.

*Bactrocera russeola* (Drew & Hancock, 1981). Asia-Pacific. Non-pest. Cue-lure.

*Bactrocera rutengiae* Drew & Romig, 2013. Asia-Pacific. Non-pest. Methyl eugenol.

*Bactrocera rutila* (Hering, 1941). Asia-Pacific. Non-pest.

*Bactrocera samoae* Drew, 1989. Asia-Pacific. Non-pest.

*Bactrocera sapaensis* Drew & Romig, 2013. Asia-Pacific. Non-pest. Cue-lure.

*Bactrocera satanellus* (Hering, 1941). Asia-Pacific. Non-pest.

*Bactrocera seguyi* (Hering, 1939). Asia-Pacific. Non-pest. Methyl eugenol.

*Bactrocera selenophora* Tsuruta & White, 2001. Asia-Pacific. Non-pest. Cue-lure.

*Bactrocera sembaliensis* Drew & Hancock, 1994. Asia-Pacific. Non-pest. Cue-lure.

*Bactrocera setinervis* (Malloch, 1938). Asia-Pacific. Non-pest.

*Bactrocera silvicola* (May, 1962). Asia-Pacific. Non-pest. Cue-lure, zingerone.

*Bactrocera simulata* (Malloch, 1939). Asia-Pacific. Non-pest. Cue-lure.

*Bactrocera speculifer* (Walker, 1865). Asia-Pacific. Fruit pest (monophagous). Methyl eugenol.

*Bactrocera speewahensis* Fay & Hancock, 2006. Asia-Pacific. Non-pest. Zingerone.

*Bactrocera splendida* (Perkins, 1938). Asia-Pacific. Non-pest.

*Bactrocera strigata* (Perkins, 1934). Asia-Pacific. Non-pest.

*Bactrocera sulawesiae* Drew & Hancock, 1994. Asia-Pacific. Non-pest. Methyl eugenol.

*Bactrocera suliae* Drew & Romig, 2013. Asia-Pacific. Non-pest. Methyl eugenol.

*Bactrocera sumbawaensis* Drew & Hancock, 1994. Asia-Pacific. Non-pest. Cue-lure.

*Bactrocera superba* Drew & Romig, 2013. Asia-Pacific. Non-pest. Cue-lure.

*Bactrocera symplocos* Drew & Romig, 2013. Asia-Pacific. Non-pest.

*Bactrocera syzygii* White & Tsuruta, 2001. Asia-Pacific. Non-pest. Zingerone.

Notes: Zingerone is a new lure record.

*Bactrocera tapahensis* Drew & Romig, 2013. Asia-Pacific. Non-pest. Methyl eugenol.

*Bactrocera tenuifascia* (May, 1965). Asia-Pacific. Non-pest. Methyl eugenol.

*Bactrocera terminaliae* Drew, 1989. Asia-Pacific. Non-pest.

*Bactrocera terminifer* (Walker, 1860). Asia-Pacific. Non-pest.

*Bactrocera ternatiae* Drew & Romig, 2013. Asia-Pacific. Non-pest. Methyl eugenol.

*Bactrocera tetrachaeta* (Bezzi, 1919). Asia-Pacific. Non-pest.

*Bactrocera thailandica* Drew & Hancock, 1994. Asia-Pacific. Non-pest. Cue-lure.

*Bactrocera thistletoni* Drew, 1989. Asia-Pacific. Non-pest. Cue-lure.

*Bactrocera tigrina* (May, 1953). Asia-Pacific. Non-pest. Zingerone.

*Bactrocera tillyardi* (Perkins, 1938). Asia-Pacific. Non-pest.

*Bactrocera tinomiscii* Drew, 1989. Asia-Pacific. Non-pest. Cue-lure.

*Bactrocera torresiae* Huxam & Hancock, 2006. Asia-Pacific. Non-pest. Cue-lure.

*Bactrocera tortuosa* White & Evenhuis, 1999. Asia-Pacific. Non-pest.

*Bactrocera toxopeusi* (Hering, 1953). Asia-Pacific. Non-pest.

*Bactrocera trifaria* (Drew, 1971). Asia-Pacific. Non-pest. Cue-lure.

*Bactrocera trifasciata* (Hardy, 1982). Asia-Pacific. Non-pest. Cue-lure.

*Bactrocera trilineola* Drew, 1989. Asia-Pacific. Fruit pest. Cue-lure.

*Bactrocera trivialis* (Drew, 1971). Asia-Pacific. Fruit pest. Cue-lure, zingerone.

*Bactrocera truncata* Drew & Romig, 2013. Asia-Pacific. Non-pest. Cue-lure.

*Bactrocera tryoni* (Froggatt, 1897). Asia-Pacific. Fruit pest. Cue-lure, zingerone.

Notes: See under *B. aquilonis*.

*Bactrocera tsuneonis* (Miyake, 1919). Asia-Pacific. Fruit pest.

*Bactrocera tuberculata* (Bezzi, 1916). Asia-Pacific. Fruit pest (polyphagous). Methyl eugenol.

*Bactrocera turneri* Drew, 1989. Asia-Pacific. Non-pest. Cue-lure.

*Bactrocera umbrosa* (Fabricius, 1805). Asia-Pacific. Fruit pest (monophagous). Methyl eugenol.

*Bactrocera unifasciata* (Malloch, 1939). Asia-Pacific. Non-pest. Cue-lure.

*Bactrocera unilineata* Drew, 1989. Asia-Pacific. Non-pest. Cue-lure.

*Bactrocera unimacula* Drew & Hancock, 1994. Asia-Pacific. Non-pest. Methyl eugenol.

*Bactrocera unipunctata* (Malloch, 1939). Asia-Pacific. Non-pest.

*Bactrocera unistriata* (Drew, 1971). Asia-Pacific. Non-pest. Methyl eugenol.

*Bactrocera unitaeniola* Drew & Romig, 2001. Asia-Pacific. Non-pest. Cue-lure.

*Bactrocera usitata* Drew & Hancock, 1994. Asia-Pacific. Non-pest. Cue-lure.

*Bactrocera ustulata* Drew, 1989. Asia-Pacific. Non-pest. Cue-lure.

*Bactrocera uvariae* Drew, 2011. Asia-Pacific. Non-pest. Cue-lure.

*Bactrocera venefica* (Hering, 1938). Asia-Pacific. Non-pest.

*Bactrocera verbascifoliae* Drew & Hancock, 1994. Asia-Pacific. Non-pest. Methyl eugenol.

*Bactrocera versicolor* (Bezzi, 1916). Asia-Pacific. Fruit pest (monophagous). Methyl eugenol.

*Bactrocera visenda* (Hardy, 1951). Asia-Pacific. Non-pest. Methyl eugenol.

*Bactrocera vishnu* Drew & Hancock, 1994. Asia-Pacific. Non-pest. Cue-lure.

*Bactrocera vulgaris* (Drew, 1971). Asia-Pacific. Non-pest. Cue-lure.

*Bactrocera waaiae* Drew & Romig, 2013. Asia-Pacific. Non-pest. Methyl eugenol.

*Bactrocera wanangiae* Drew & Hancock, 2016. Asia-Pacific. Non-pest.

*Bactrocera warisensis* White & Evenhuis, 1999. Asia-Pacific. Non-pest.

*Bactrocera wuzhishana* Li & Wang, 2006. Asia-Pacific. Non-pest. Methyl eugenol.

*Bactrocera xanthodes* (Broun, 1904). Asia-Pacific. Fruit pest. Methyl eugenol.

*Bactrocera yayeyamana* (Matsumara, 1916). Asia-Pacific. Non-pest.

*Bactrocera yorkensis* Drew & Hancock, 1999. Asia-Pacific. Non-pest. Methyl eugenol.

*Bactrocera zonata* (Saunders, 1842). Asia-Pacific. Fruit pest (polyphagous). Methyl eugenol.

Notes: Natively distributed in Asia, from the Indian subcontinent to Vietnam, and invasive in the Afrotropical and West-Palearctic (Middle East) Regions.

**Genus *Dacus* Fabricius**

*Dacus abbabae* Munro, 1933. Africa. Non-pest.

*Dacus abditus* (Munro, 1984). Africa. Non-pest.

*Dacus abruptus* White, 2009. Africa. Non-pest.

*Dacus absonifacies* (May, 1956). Asia-Pacific. Non-pest. Cue-lure, zingerone.

*Dacus acutus* White, 2009. Africa. Non-pest.

*Dacus adenae* (Hering, 1940). Africa. Non-pest.

*Dacus adenionis* Munro, 1984. Africa. Non-pest.

*Dacus adustus* Munro, 1948. Africa. Non-pest.

*Dacus aequalis* Coquillett, 1909. Asia-Pacific. Non-pest. Cue-lure, zingerone.

*Dacus africanus* Adams, 1905. Africa. Non-pest. Cue-lure.

*Dacus alarifumidus* Drew, 1989. Asia-Pacific. Non-pest. Cue-lure.

*Dacus albiseta* White, 2009. Africa. Non-pest. Cue-lure.

*Dacus alulapictus* Drew, 1989. Asia-Pacific. Non-pest.

*Dacus amberiens* (Munro, 1984). Africa. Non-pest.

*Dacus ambonensis* Drew & Hancock, 1998. Asia-Pacific. Non-pest. Cue-lure.

*Dacus amphoratus* (Munro, 1984). Africa. Non-pest.

*Dacus aneuvittatus* (Drew, 1971). Asia-Pacific. Non-pest.

*Dacus annulatus* Becker, 1903. Africa. Non-pest.

*Dacus apectus* White, 2006. Africa. Non-pest.

*Dacus apiculatus* White, 2006. Africa. Non-pest. Cue-lure.

*Dacus apostata* (Hering, 1937). Africa. Non-pest.

*Dacus apoxanthus* Bezzi, 1924. Africa. Non-pest.

*Dacus arabicus* White, 2006. Africa. Non-pest.

*Dacus arcuatus* Munro, 1939. Africa. Non-pest.

*Dacus armatus* Fabricius, 1805. Africa. Non-pest. Cue-lure.

Notes: Type species for the genus.

*Dacus aspilus* Bezzi, 1924. Africa. Non-pest.

*Dacus atrimarginatus* Drew & Hancock, 1998. Asia-Pacific. Non-pest.

*Dacus attenuatus* Collart, 1935. Africa. Non-pest.

*Dacus axanthinus* White & Evenhuis, 1999. Asia-Pacific. Non-pest.

*Dacus axanus* (Hering 1938). Asia-Pacific. Cucurbitaceae fruit pest. Cue-lure, zingerone.

Notes: *Dacus axanus* is a species that is common in Australia and Papua New Guinea, and this name appears in various pest-related resources. *D. unicolor* and *D. vespiformis* may be conspecific with *D. axanus*. The latter two were described in a single publication by Hendel (Hendel 1927) without illustrations and the descriptions do not differentiate either from *D. axanus*. The types of *D. unicolor* and *D. vespiformis* were lost during the fire at the Museum in Hamburg in 1943.

*Dacus badius* Drew, 1989. Asia-Pacific. Non-pest. Cue-lure.

*Dacus bakingiliensis* Hancock, 1985. Africa. Non-pest. Cue-lure.

*Dacus bannatus* Wang, 1990. Asia-Pacific. Non-pest. Cue-lure.

*Dacus basifasciatus* (Hering, 1941). Africa. Non-pest.

*Dacus bellulus* Drew & Hancock, 1981. Asia-Pacific. Non-pest. Cue-lure, zingerone.

*Dacus bequaerti* Collart, 1935. Africa. Non-pest.

*Dacus bidens* (Curran, 1927). Africa. Non-pest.

*Dacus binotatus* Loew, 1862. Africa. Non-pest. Cue-lure.

*Dacus bispinosus* (Wang, 1990). Asia-Pacific. Non-pest.

*Dacus bistrigulatus* Bezzi, 1908. Africa. Non-pest.

*Dacus bivittatus* (Bigot, 1858). Africa. Cucurbitaceae fruit pest. Cue-lure.

*Dacus blepharogaster* Bezzi, 1917. Africa. Non-pest.

*Dacus bombastus* Hering, 1941. Africa. Non-pest.

*Dacus botianus* (Munro, 1984). Africa. Non-pest.

*Dacus brevis* Coquillett, 1901. Africa. Non-pest.

*Dacus brevistriga* Walker, 1861. Africa. Non-pest.

*Dacus briani* White, 2006. Africa. Non-pest.

*Dacus brunnalis* White, 2009. Africa. Non-pest.

*Dacus calirayae* Drew & Hancock, 1998. Asia-Pacific. Non-pest. Cue-lure.

*Dacus capillaris* (Drew, 1972). Asia-Pacific. Non-pest. Cue-lure.

*Dacus carnesi* (Munro, 1984). Africa. Non-pest.

*Dacus carvalhoi* (Munro, 1984). Africa. Non-pest.

*Dacus ceropegiae* (Munro, 1984). Africa. Non-pest.

*Dacus chamun* (Munro, 1984). Africa. Non-pest.

*Dacus chapini* Curran, 1927. Africa. Non-pest.

*Dacus chiwira* Hancock, 1985. Africa. Non-pest. Cue-lure.

*Dacus chrysomphalus* (Bezzi, 1924). Africa. Non-pest. Cue-lure.

*Dacus ciliatus* Loew, 1862 Africa. Cucurbitaceae fruit pest.

Notes: Native to the Aftrotropical Region, and invasive in the Middle East and the Indian subcontinent (India, Pakistan, Bangladesh, Sri Lanka).

*Dacus clinophlebs* Hendel, 1928. Africa. Non-pest.

*Dacus coenensis* Royer & Hancock, 2012. Asia-Pacific. Non-pest. Cue-lure.

*Dacus collarti* Munro, 1938. Africa. Non-pest.

*Dacus congoensis* White, 2006. Africa. Non-pest.

*Dacus conopsoides* de Meijere, 1911. Asia-Pacific. Non-pest.

*Dacus copelandi* White, 2006. Africa. Non-pest.

*Dacus crabroniformis* (Bezzi, 1914). Asia-Pacific. Non-pest.

*Dacus croceus* Munro, 1957. Africa. Non-pest.

*Dacus cyathus* (Munro, 1984). Africa. Non-pest.

*Dacus delicatus* Munro, 1939. Africa. Non-pest.

*Dacus deltatus* White, 2006. Africa. Non-pest.

*Dacus demmerezi* (Bezzi, 1917). Africa. Cucurbitaceae fruit pest. Cue-lure.

*Dacus devure* Hancock, 1985. Africa. Non-pest. Cue-lure.

*Dacus diastatus* Munro, 1984. Africa. Non-pest. Cue-lure.

*Dacus discipennis* (Walker, 1861). Asia-Pacific. Non-pest.

*Dacus discophorus* (Hering, 1956). Asia-Pacific. Non-pest. Cue-lure.

*Dacus discors* Drew, 1989. Asia-Pacific. Non-pest. Cue-lure.

*Dacus discretus* Drew & Romig, 2013. Asia-Pacific. Non-pest. Cue-lure.

*Dacus disjunctus* (Bezzi, 1915). Africa. Non-pest.

*Dacus dissimilis* Drew, 1989. Asia-Pacific. Non-pest. Cue-lure.

*Dacus donggaliae* Drew & Romig, 2013. Asia-Pacific. Non-pest. Cue-lure.

*Dacus dorjii* Drew & Romig, 2007. Asia-Pacific. Non-pest. Cue-lure.

*Dacus durbanensis* Munro, 1935. Africa. Non-pest. Cue-lure, zingerone.

Notes: The attraction to zingerone was documented by Manrakhan et al. (2017).

*Dacus eclipsis* (Bezzi, 1924). Africa. Non-pest. Cue-lure.

*Dacus elatus* White, 2006. Africa. Non-pest.

*Dacus elegans* (Munro, 1984). Africa. Non-pest.

*Dacus elutissimus* Bezzi, 1924. Africa. Non-pest.

*Dacus eminus* Munro 1939. Africa. Non-pest. Cue-lure.

*Dacus erythraeus* Bezzi, 1917. Africa. Non-pest.

*Dacus esakii* (Shiraki, 1939). Asia-Pacific. Non-pest.

*Dacus etiennellus* Munro, 1984. Africa. Non-pest. Cue-lure.

*Dacus externellus* (Munro, 1984). Africa. Non-pest.

*Dacus famona* Hancock, 1985. Africa. Non-pest. Cue-lure.

*Dacus fasciolatus* Collart, 1940. Africa. Non-pest.

*Dacus feijeni* White, 1998. Asia-Pacific. Non-pest. Cue-lure.

*Dacus ficicola* Bezzi, 1915. Africa. Non-pest.

*Dacus fissuratus* White, 2006. Africa. Non-pest.

*Dacus flavicrus* Graham, 1910. Africa. Non-pest.

*Dacus fletcheri* Drew & Romig, 2007. Asia-Pacific. Non-pest. Cue-lure.

*Dacus formosanus* (Tseng & Chu, 1983). Asia-Pacific. Non-pest. Cue-lure.

*Dacus freidbergi* (Munro, 1984). Africa. Non-pest.

*Dacus frontalis* Becker, 1922. Africa. Cucurbitaceae fruit pest. Cue-lure, zingerone.

Notes: The attraction to zingerone was documented by Manrakhan et al. (2017).

*Dacus fumosus* Collart, 1935. Africa. Non-pest.

*Dacus fuscatus* Wiedemann, 1819. Africa. Non-pest.

*Dacus fuscinervis* Malloch, 1932. Africa. Non-pest.

*Dacus fuscovittatus* Graham, 1910. Africa. Non-pest. Cue-lure.

*Dacus gabonensis* White, 2006. Africa. Non-pest.

*Dacus ghesquierei* Collart, 1935. Africa. Non-pest.

*Dacus goergeni* De Meyer, White & Goodger, 2013. Africa. Non-pest.

*Dacus guineensis* Hering, 1944. Africa. Non-pest.

*Dacus gypsoides* Munro, 1933. Africa. Non-pest.

*Dacus hainanus* Wang & Zhao, 1989. Asia-Pacific. Non-pest.

*Dacus hamatus* Bezzi, 1917. Africa. Non-pest.

*Dacus hapalus* (Munro, 1984). Africa. Non-pest.

*Dacus hardyi* Drew, 1979. Asia-Pacific. Non-pest. Cue-lure.

*Dacus hargreavesi* (Munro, 1939). Africa. Non-pest.

*Dacus herensis* (Munro, 1984). Africa. Non-pest.

*Dacus humeralis* (Bezzi, 1915). Africa. Non-pest. Cue-lure.

*Dacus hyalobasis* Bezzi, 1924. Africa. Non-pest.

*Dacus iaspideus* Munro, 1948. Africa. Non-pest.

*Dacus icariiformis* (Enderlein, 1920). Asia-Pacific. Non-pest.

*Dacus ikelenge* Hancock, 1985. Africa. Non-pest. Cue-lure.

*Dacus impar* Drew, 1989. Asia-Pacific. Non-pest.

*Dacus inclytus* (Munro, 1984). Africa. Non-pest.

*Dacus indecorus* (Hardy, 1974). Asia-Pacific. Non-pest.

*Dacus infernus* (Hardy, 1973). Asia-Pacific. Non-pest.

*Dacus inflatus* Munro, 1939. Africa. Non-pest.

*Dacus inornatus* Bezzi, 1908. Africa. Non-pest.

*Dacus insolitus* White, 2009. Africa. Non-pest.

*Dacus insulosus* Drew & Hancock, 1998. Asia-Pacific. Non-pest.

*Dacus jubatus* (Munro, 1984). Africa. Non-pest.

*Dacus kakamega* White, 2006. Africa. Non-pest.

*Dacus kaplanae* White, 2009. Africa. Non-pest.

*Dacus kariba* Hancock, 1985. Africa. Non-pest. Cue-lure.

*Dacus katonae* Bezzi, 1924. Africa. Non-pest.

*Dacus keiseri* (Hering, 1956). Asia-Pacific. Non-pest.

*Dacus kurrensis* White, 2009. Africa. Non-pest.

*Dacus lagunae* Drew & Hancock, 1998. Asia-Pacific. Non-pest. Cue-lure.

*Dacus langi* Curran, 1927. Africa. Non-pest. Cue-lure.

*Dacus leongi* Drew & Hancock, 1998. Asia-Pacific. Non-pest. Cue-lure.

*Dacus limbipennis* Macquart, 1843. Africa. Cucurbitaceae fruit pest.

*Dacus linearis* Collart, 1935. Africa. Non-pest.

*Dacus longicornis* (Wiedemann, 1830). Asia-Pacific. Cucurbitaceae fruit pest. Cue-lure.

*Dacus longistylus* Wiedemann, 1830. Africa. Non-pest.

*Dacus lotus* (Bezzi, 1924). Africa. Non-pest.

*Dacus lounsburyii* Coquillett, 1901. Africa. Cucurbitaceae fruit pest.

*Dacus luteovittatus* White, 2009. Africa. Non-pest.

*Dacus macer* Bezzi, 1919. Africa. Non-pest.

*Dacus maculipterus* Drew & Hancock, 1998. Asia-Pacific. Non-pest.

*Dacus madagascarensis* White, 2006. Africa. Non-pest. Cue-lure.

*Dacus magnificus* White, 2009. Africa. Non-pest.

*Dacus maprikensis* Drew, 1989. Asia-Pacific. Non-pest.

*Dacus marshalli* Bezzi, 1924. Africa. Non-pest.

*Dacus masaicus* Munro, 1937. Africa. Non-pest. Cue-lure.

*Dacus mayi* (Drew, 1972). Asia-Pacific. Non-pest. Cue-lure.

*Dacus maynei* Bezzi, 1924. Africa. Non-pest.

*Dacus mediovittatus* White, 2006. Africa. Non-pest. Cue-lure.

*Dacus meladassus* (Munro, 1984). Africa. Non-pest.

*Dacus melanaspis* (Munro, 1984). Africa. Non-pest.

*Dacus melanohumeralis* Drew, 1989. Asia-Pacific. Non-pest. Methyl eugenol.

*Dacus melanopectus* Drew & Romig, 2013. Asia-Pacific. Non-pest. Methyl eugenol.

*Dacus merzi* White, 2006. Africa. Non-pest.

*Dacus mirificus* (Munro, 1984). Africa. Non-pest.

*Dacus mochii* Bezzi, 1917. Africa. Non-pest.

*Dacus mulgens* Munro, 1932. Africa. Non-pest.

*Dacus murphyi* Drew & Hancock, 1998. Asia-Pacific. Non-pest.

*Dacus nairobensis* White, 2006. Africa. Non-pest.

*Dacus namibiensis* Hancock & Drew, 2001. Africa. Non-pest.

*Dacus nanggalae* Drew & Hancock, 1998. Asia-Pacific. Non-pest. Cue-lure.

*Dacus nanus* Collart, 1940. Africa. Non-pest.

*Dacus newmani* (Perkins, 1937). Asia-Pacific. Non-pest. Cue-lure.

*Dacus nigriscutatus* White, 2006. Africa. Non-pest.

*Dacus nigrolateris* White, 2006. Africa. Non-pest.

*Dacus notalaxus* Munro, 1984. Africa. Non-pest.

*Dacus nummularius* (Bezzi, 1916). Asia-Pacific. Non-pest. Cue-lure.

*Dacus obesus* Munro, 1948. Africa. Non-pest.

*Dacus okumuae* White, 2006. Africa. Non-pest.

*Dacus ooii* Drew & Hancock, 1998. Asia-Pacific. Non-pest. Cue-lure.

*Dacus opacatus* Munro, 1948. Africa. Non-pest.

*Dacus ortholomatus* Hardy, 1982. Asia-Pacific. Non-pest.

*Dacus ostiofaciens* Munro, 1932. Africa. Non-pest.

*Dacus pallidilatus* Munro, 1948. Africa. Non-pest. Cue-lure.

*Dacus palmerensis* Drew, 1989. Asia-Pacific. Non-pest. Cue-lure.

*Dacus pamelae* (Munro, 1984). Africa. Non-pest.

*Dacus panpyrrhus* (Munro, 1984). Africa. Non-pest.

*Dacus parvimaculatus* White, 2006. Africa. Non-pest.

*Dacus pecropsis* Munro, 1984. Africa. Non-pest. Cue-lure.

*Dacus pedunculatus* (Bezzi, 1919). Asia-Pacific. Non-pest.

*Dacus pergulariae* Munro, 1938. Africa. Non-pest.

*Dacus persicus* Hendel, 1927. Asia-Pacific. Non-pest.

*Dacus petioliforma* (May, 1956). Asia-Pacific. Non-pest. Cue-lure.

*Dacus phantoma* Hering, 1941. Africa. Non-pest.

*Dacus phimis* (Munro, 1984). Africa. Non-pest.

*Dacus phloginus* (Munro, 1984). Africa. Non-pest.

*Dacus pictus* (Hardy, 1970). Asia-Pacific. Non-pest.

*Dacus plagiatus* Collart, 1935. Africa. Non-pest.

*Dacus pleuralis* Collart, 1935. Africa. Non-pest. Cue-lure.

*Dacus polistiformis* (Senior-White, 1922). Asia-Pacific. Non-pest.

*Dacus pseudapostata* White, 2009. Africa. Non-pest.

*Dacus pseudomirificus* White, 2009. Africa. Non-pest.

*Dacus pulchralis* White, 2006. Africa. Non-pest. Cue-lure.

*Dacus pullescens* Munro, 1948. Africa. Non-pest.

*Dacus pullus* (Hardy, 1982). Asia-Pacific. Non-pest.

*Dacus punctatifrons* Karsch, 1887. Africa. Cucurbitaceae fruit pest. Cue-lure.

*Dacus purpurifrons* Bezzi, 1924. Africa. Non-pest.

*Dacus purus* (Curran, 1927). Africa. Non-pest.

*Dacus pusillator* (Munro, 1984). Africa. Non-pest.

*Dacus pusillus* (May, 1965). Asia-Pacific. Non-pest. Methyl eugenol.

*Dacus quilicii* White, 2006. Africa. Non-pest. Cue-lure.

*Dacus radmirus* Hering, 1941. Africa. Non-pest.

*Dacus ramanii* Drew & Hancock, 1998. Asia-Pacific. Non-pest. Cue-lure.

*Dacus rubicundus* Bezzi, 1924. Africa. Non-pest.

*Dacus rufoscutellatus* (Hering, 1937). Africa. Non-pest.

*Dacus rufus* Bezzi, 1915. Africa. Non-pest.

*Dacus rugatus* Munro, 1984. Africa. Non-pest.

*Dacus ruslan* (Hering, 1941). Africa. Non-pest.

*Dacus rutilus* Munro, 1948. Africa. Non-pest.

*Dacus sakeji* Hancock, 1985. Africa. Non-pest. Cue-lure.

*Dacus salamander* (Drew & Hancock, 1981). Asia-Pacific. Non-pest. Cue-lure.

*Dacus santongae* Drew & Hancock, 1998. Asia-Pacific. Non-pest. Cue-lure.

*Dacus satanas* (Hering, 1939). Asia-Pacific. Non-pest. Zingerone.

Notes: Zingerone is a new lure record.

*Dacus scaber* Loew, 1862. Africa. Non-pest.

*Dacus schoutedeni* Collart, 1935. Africa. Non-pest.

*Dacus secamoneae* Drew, 1989. Asia-Pacific. Non-pest. Cue-lure, zingerone.

*Dacus segunii* White, 2006. Africa. Non-pest. Cue-lure.

*Dacus seguyi* (Munro, 1984). Africa. Non-pest.

*Dacus semisphaereus* Becker, 1903. Africa. Non-pest.

*Dacus senegalensis* White, 2009. Africa. Non-pest.

*Dacus serratus* (Munro, 1984). Africa. Non-pest.

*Dacus setilatens* Munro, 1984. Africa. Non-pest.

*Dacus siamensis* Drew & Hancock, 1998. Asia-Pacific. Non-pest. Cue-lure.

*Dacus signatifrons* (May, 1956). Asia-Pacific. Non-pest. Cue-lure.

*Dacus siliqualactis* Munro, 1939. Africa. Non-pest.

*Dacus sinensis* Wang, 1990. Asia-Pacific. Non-pest.

*Dacus solomonensis* Malloch, 1939. Asia-Pacific. Cucurbitaceae fruit pest. Cue-lure.

*Dacus sphaeristicus* Speiser, 1910. Africa. Non-pest.

*Dacus sphaeroidalis* (Bezzi, 1916). Asia-Pacific. Non-pest. Cue-lure.

*Dacus sphaerostigma* (Bezzi, 1924). Africa. Non-pest.

*Dacus spissus* Munro, 1984. Africa. Non-pest.

*Dacus stentor* Munro, 1929. Africa. Non-pest.

*Dacus stylifer* (Bezzi, 1919). Africa. Non-pest.

*Dacus subsessilis* (Bezzi, 1919). Asia-Pacific. Non-pest.

*Dacus succaelestis* Ito, 2011. Asia-Pacific. Non-pest.

*Dacus taui* Drew & Romig, 2001. Asia-Pacific. Non-pest. Cue-lure.

*Dacus telfaireae* (Bezzi, 1924). Africa. Non-pest. Cue-lure.

*Dacus temnopterus* Bezzi, 1928. Africa. Non-pest.

*Dacus tenebricus* Munro, 1938. Africa. Non-pest.

*Dacus tenebrosus* Drew & Hancock, 1998. Asia-Pacific. Non-pest. Cue-lure, zingerone.

Notes: Zingerone is a new lure record.

*Dacus theophrastus* Hering, 1941. Africa. Non-pest. Cue-lure.

*Dacus transitorius* Collart, 1935. Africa. Non-pest.

*Dacus transversalis* White, 2009. Africa. Non-pest.

*Dacus triater* Munro, 1937. Africa. Non-pest.

*Dacus trigonus* Bezzi, 1919. Africa. Non-pest.

*Dacus trimacula* Wang, 1990. Asia-Pacific. Non-pest. Cue-lure, zingerone.

Notes: Zingerone is a new lure record.

*Dacus triquetrus* Drew & Romig, 2013. Asia-Pacific. Non-pest. Cue-lure.

*Dacus umbeluzinus* (Munro, 1984). Africa. Non-pest.

*Dacus umbrilatus* Munro, 1938. Africa. Non-pest.

*Dacus umehi* White, 2006. Africa. Non-pest.

*Dacus unicolor* (Hendel, 1927). Asia-Pacific. Non-pest.

Notes: See under *D. axanus*

*Dacus velutifrons* White, 2009. Africa. Non-pest.

*Dacus venetatus* Munro, 1939. Africa. Non-pest. Cue-lure.

*Dacus vertebratus* Bezzi, 1908. Africa. Cucurbitaceae fruit pest. Cue-lure.

*Dacus vespiformis* (Hendel, 1927). Asia-Pacific. Non-pest.

Notes: See under *D. axanus*.

*Dacus vestigivittatus* White, 2009. Africa. Non-pest.

*Dacus viator* Munro, 1939. Africa. Non-pest.

*Dacus vijaysegarani* Drew & Hancock, 1998. Asia-Pacific. Non-pest. Cue-lure, zingerone.

Notes: Zingerone is a new lure record.

*Dacus vittatus* (Hardy, 1974). Asia-Pacific. Non-pest.

*Dacus wallacei* White, 1998. Asia-Pacific. Non-pest.

*Dacus woodi* Bezzi, 1917. Africa. Non-pest.

*Dacus xanthaspis* (Munro, 1984). Africa. Non-pest.

*Dacus xanthinus* White, 2009. Africa. Non-pest.

*Dacus xanthopterus* (Bezzi, 1915). Africa. Non-pest. Cue-lure.

*Dacus xanthopus* Bezzi, 1924. Africa. Non-pest.

*Dacus yangambinus* Munro, 1984. Africa. Non-pest.

*Dacus yaromi* White, 2009. Africa. Non-pest.

*Dacus yemenensis* White, 2006. Africa. Non-pest.

**Genus *Monacrostichus* Bezzi**

*Monacrostichus citricola* (Bezzi, 1913). Asia-Pacific. Fruit pest.

Notes: Type species for the genus.

*Monacrostichus malaysiae* Drew & Hancock, 1994. Asia-Pacific. Non-pest.

**Genus *Zeugodacus* Hendel**

*Zeugodacus abdoangustus* (Drew, 1972). Asia-Pacific. Non-pest. Cue-lure.

*Zeugodacus abdoaurantiacus* (Drew, 1989). Asia-Pacific. Non-pest.

*Zeugodacus abdopallescens* (Drew, 1971). Asia-Pacific. Non-pest. Cue-lure.

*Zeugodacus ablepharus* (Bezzi, 1919). Asia-Pacific. Non-pest.

*Zeugodacus abnormis* (Hardy, 1982). Asia-Pacific. Non-pest. Cue-lure.

*Zeugodacus absolutus* (Walker, 1861). Asia-Pacific. Non-pest.

*Zeugodacus aithonota* (Drew & Romig, 2013). Asia-Pacific. Non-pest. Cue-lure.

*Zeugodacus alampetus* (Drew, 1989). Asia-Pacific. Non-pest. Methyl eugenol.

*Zeugodacus ambiguus* (Shiraki, 1933). Asia-Pacific. Non-pest. Cue-lure.

*Zeugodacus amoenus* (Drew, 1972). Asia-Pacific. Non-pest. Cue-lure.

*Zeugodacus anala* (Chen & Zhou, 2013). Asia-Pacific. Non-pest.

Notes: We regard this name as a noun, not changing the ending, following De Meyer et al. (2015). Chen and Zhou (2013) did not specify if it was meant as a noun or adjective, but mentioned “the specific ephithet refers to the wing anal streak”.

*Zeugodacus anchitrichotus* (Drew, 1989). Asia-Pacific. Non-pest.

*Zeugodacus angusticostatus* (Drew, 1989). Asia-Pacific. Non-pest. Cue-lure.

*Zeugodacus angustifinis* (Hardy, 1982). Asia-Pacific. Non-pest. Cue-lure.

*Zeugodacus apicalis* (de Meijere, 1911). Asia-Pacific. Non-pest. Cue-lure.

*Zeugodacus apiciflavus* (Yu He & Chen, 2011). Asia-Pacific. Non-pest.

*Zeugodacus apicofemoralis* (Drew & Romig, 2013). Asia-Pacific. Non-pest. Cue-lure.

*Zeugodacus areolatus* (Walker, 1861). Asia-Pacific. Non-pest.

*Zeugodacus arisanicus* Shiraki, 1933, stat. rev. Asia-Pacific. Non-pest. Cue-lure.

Notes: This species is here reassigned to *Zeugodacus*. It has a medial postsutural vitta and yellow markings anterior of the transverse suture, which are likely reliable morphological characters for assignment to *Zeugodacus*. This generic assignment is further supported by DNA sequence data from seven genes (San Jose et al. 2018). Whether the other members assigned to the subgenus Hemizeugodacus should be placed in *Bactrocera* or *Zeugodacus* remains to be determined.

*Zeugodacus armillatus* (Hering, 1938). Asia-Pacific. Non-pest.

*Zeugodacus assamensis* White, 1999. Asia-Pacific. Non-pest. Cue-lure.

*Zeugodacus atrichus* (Bezzi, 1919). Asia-Pacific. Non-pest.

*Zeugodacus atrifacies* (Perkins, 1938). Asia-Pacific. Non-pest. Cue-lure.

*Zeugodacus atrisetosus* (Perkins, 1939). Asia-Pacific. Cucurbitaceae fruit pest.

*Zeugodacus atypicus* (White & Evenhuis, 1999). Asia-Pacific. Non-pest.

*Zeugodacus aurantiventer* (Drew, 1989). Asia-Pacific. Non-pest. Cue-lure.

*Zeugodacus bakeri* (Bezzi, 1919). Asia-Pacific. Non-pest.

*Zeugodacus baliensis* (Drew & Romig, 2013). Asia-Pacific. Non-pest. Cue-lure.

*Zeugodacus baoshanensis* (Zhang, Ji, Yang & Chen, 2011). Asia-Pacific. Non-pest.

*Zeugodacus biguttatus* (Bezzi, 1916). Asia-Pacific. Non-pest. Cue-lure.

*Zeugodacus binoyi* (Drew, 2002). Asia-Pacific. Non-pest. Cue-lure.

*Zeugodacus bogorensis* (Hardy, 1983). Asia-Pacific. Non-pest. Cue-lure.

*Zeugodacus borongensis* (Drew & Romig, 2013). Asia-Pacific. Non-pest. Cue-lure.

*Zeugodacus brachus* (Drew, 1972). Asia-Pacific. Non-pest. Cue-lure.

*Zeugodacus brevipunctatus* (David & Hancock, 2017), comb. n. Asia-Pacific. Non-pest. Cue-lure.

Notes: This species was recently described in *Bactrocera* and placed in the subgenus Sinodacus, of which all other previous members have been transferred to *Zeugodacus* (De Meyer et al. 2015). We here follow this reasoning.

*Zeugodacus brevivitta* (Drew & Romig, 2013). Asia-Pacific. Non-pest.

*Zeugodacus buruensis* (White, 1999). Asia-Pacific. Non-pest. Cue-lure.

*Zeugodacus buvittatus* (Drew, 1989). Asia-Pacific. Non-pest. Cue-lure.

*Zeugodacus calumniatus* (Hardy, 1970). Asia-Pacific. Non-pest. Methyl eugenol.

*Zeugodacus careomacula* (Drew & Romig, 2013). Asia-Pacific. Non-pest. Cue-lure.

*Zeugodacus caudatus* (Fabricius, 1805). Asia-Pacific. Cucurbitaceae flower pest. Cue-lure.

Notes: Type species for genus.

*Zeugodacus choristus* (May, 1962). Asia-Pacific. Non-pest. Cue-lure.

*Zeugodacus cilifer* (Hendel, 1912). Asia-Pacific. Non-pest. Cue-lure.

*Zeugodacus citrifuscus* (Drew & Romig, 2013). Asia-Pacific. Non-pest.

*Zeugodacus citroides* (Drew, 1989). Asia-Pacific. Non-pest. Cue-lure.

*Zeugodacus complicatus* (White, 1999). Asia-Pacific. Non-pest. Cue-lure.

*Zeugodacus connexus* (Hardy, 1982). Asia-Pacific. Non-pest.

*Zeugodacus cucumis* (French, 1907). Asia-Pacific. Cucurbitaceae fruit pest.

*Zeugodacus cucurbitae* (Coquillett, 1899). Asia-Pacific. Cucurbitaceae fruit pest. Cue-lure, zingerone.

Notes: *Zeugodacus cucurbitae*, the melon fly, is one of the most significant pest species with the Tephritidae. Although different forms are recognized that can be correlated with different hosts, these are generally not thought to represent different (cryptic) species (De Meyer et al. 2015, Hendrichs et al. 2015). Natively widespread in Asia and invasive in many Pacific islands and the Afrotropical region.

*Zeugodacus curtus* (Drew, 1972). Asia-Pacific. Non-pest. Cue-lure.

*Zeugodacus daclaciae* (Drew & Romig, 2013). Asia-Pacific. Non-pest. Cue-lure.

*Zeugodacus daulus* (Drew, 1989). Asia-Pacific. Non-pest. Cue-lure.

*Zeugodacus decipiens* (Drew, 1972). Asia-Pacific. Cucurbitaceae fruit pest.

*Zeugodacus depressus* (Shiraki, 1933). Asia-Pacific. Cucurbitaceae fruit pest.

*Zeugodacus diaphoropsis* (Hering, 1952). Asia-Pacific. Non-pest.

*Zeugodacus diaphorus* (Hendel, 1915). Asia-Pacific. Non-pest. Cue-lure.

*Zeugodacus dissidens* (Drew, 1989). Asia-Pacific. Non-pest.

*Zeugodacus disturgidus* (Yu, Deng & Chen, 2012). Asia-Pacific. Non-pest.

Notes: *Z. disturgidus* is not included in the Drew and Romig (2013, 2016) keys. According to the diagnosis, it is similar to *Z. vinnulus* but differs in having the face with two bands, and the costal band on the wing confluent with vein R_2+3_ and not expanded apically.

*Zeugodacus diversus* (Coquillett, 1904). Asia-Pacific. Cucurbitaceae flower pest. Methyl eugenol.

Notes: Drew and Romig (2013) state that this species appears to have a weak attraction to methyl eugenol. We hereby confirm this attraction, based on the recent capture of fifteen flies among eight different trapping locations in Nepal and additional records from Bangladesh.

*Zeugodacus dorsirufus* (Drew & Romig, 2013). Asia-Pacific. Non-pest. Cue-lure.

*Zeugodacus dubiosus* (Hardy, 1982). Asia-Pacific. Non-pest. Cue-lure.

*Zeugodacus duplicatus* (Bezzi, 1916). Asia-Pacific. Non-pest.

*Zeugodacus elegantulus* (Hardy, 1974). Asia-Pacific. Non-pest. Cue-lure.

*Zeugodacus emarginatus* (Perkins, 1939). Asia-Pacific. Non-pest.

*Zeugodacus emittens* (Walker, 1860). Asia-Pacific. Non-pest. Cue-lure.

*Zeugodacus eurylomatus* (Hardy, 1982). Asia-Pacific. Non-pest.

*Zeugodacus exornatus* (Hering, 1941). Asia-Pacific. Non-pest. Cue-lure

*Zeugodacus fallacis* (Drew, 1972). Asia-Pacific. Non-pest. Cue-lure.

*Zeugodacus fereuncinatus* (Drew & Romig, 2013). Asia-Pacific. Non-pest. Cue-lure.

*Zeugodacus flavipilosus* (Hardy, 1982). Asia-Pacific. Non-pest. Cue-lure.

*Zeugodacus flavolateralis* (Drew & Romig, 2013). Asia-Pacific. Non-pest.

*Zeugodacus flavopectoralis* (Hering, 1953). Asia-Pacific. Non-pest.

*Zeugodacus flavoverticalis* (Drew & Romig, 2013). Asia-Pacific. Non-pest. Cue-lure.

*Zeugodacus freidbergi* (White, 1999). Asia-Pacific. Non-pest.

*Zeugodacus fulvipes* (Perkins, 1938). Asia-Pacific. Non-pest.

*Zeugodacus fulvoabdominalis* (White & Evenhuis, 1999). Asia-Pacific. Non-pest.

*Zeugodacus fuscipennulus* (Drew & Romig, 2001). Asia-Pacific. Non-pest.

*Zeugodacus fuscoalatus* (Drew & Romig, 2013). Asia-Pacific. Non-pest.

*Zeugodacus gavisus* (Munro, 1935). Asia-Pacific. Non-pest. Cue-lure.

*Zeugodacus gracilis* (Drew, 1972). Asia-Pacific. Non-pest. Cue-lure.

*Zeugodacus hamaceki* (Drew & Romig, 2001). Asia-Pacific. Non-pest. Cue-lure.

*Zeugodacus hancocki* (Drew & Romig, 2013). Asia-Pacific. Non-pest. Cue-lure.

*Zeugodacus hatyaiensis* (Drew & Romig, 2013). Asia-Pacific. Non-pest. Cue-lure.

*Zeugodacus havelockiae* (Drew & Romig, 2013). Asia-Pacific. Non-pest. Cue-lure.

*Zeugodacus heinrichi* (Hering, 1941). Asia-Pacific. Non-pest. Cue-lure, zingerone.

Notes: Zingerone is a new lure record.

*Zeugodacus hekouanus* (Yu He & Yang, 2011). Asia-Pacific. Non-pest.

*Zeugodacus hengsawadae* (Drew & Romig, 2013). Asia-Pacific. Non-pest.

*Zeugodacus hoabinhiae* (Drew & Romig, 2013). Asia-Pacific. Non-pest. Cue-lure

*Zeugodacus hochii* (Zia, 1936). Asia-Pacific. Cucurbitaceae fruit pest. Cue-lure, zingerone.

Notes: Zingerone is a new lure record.

*Zeugodacus hodgsoniae* (Drew & Romig, 2013). Asia-Pacific. Non-pest.

*Zeugodacus hoedi* (White, 1999). Asia-Pacific. Non-pest.

*Zeugodacus hululangatiae* (Drew & Romig, 2013). Asia-Pacific. Non-pest. Cue-lure.

*Zeugodacus incisus* (Walker, 1861). Asia-Pacific. Non-pest. Cue-lure.

*Zeugodacus indentus* (Hardy, 1974). Asia-Pacific. Non-pest.

*Zeugodacus infestus* (Enderlein, 1920). Asia-Pacific. Non-pest. Cue-lure.

*Zeugodacus iriomotiae* (Drew & Romig, 2013). Asia-Pacific. Non-pest. Methyl eugenol.

*Zeugodacus ishigakiensis* (Shiraki, 1933). Asia-Pacific. Non-pest. Cue-lure.

*Zeugodacus isolatus* (Hardy, 1973). Asia-Pacific. Non-pest. Cue-lure.

*Zeugodacus javadicus* (Mahmood, 1999). Asia-Pacific. Non-pest.

*Zeugodacus javanensis* (Perkins, 1938), comb. n. Asia-Pacific. Non-pest.

Notes: Originally described in *Afrodacus*, here transferred from *Bactrocera*. It is placed in the subgenus Javadacus. Members of *Javadacus* were not moved to *Zeugodacus* by De Meyer et al. (2015) because only one representative, *B. unirufa* Drew, 1989, had been included in any molecular phylogenetic studies, where it was robustly placed in *Bactrocera*. However, *B. unirufa* has since been synonymized with *B. melanothoracica* and removed from *Javadacus* along with several other species that did not have the shallow posterior emargination of sternite V and elongate posterior surstylus lobes in the male genitalia, which fit *Zeugodacus*. We therefore now move all remaining species in the subgenus Javadacus to *Zeugodacus*.

*Zeugodacus juxtuncinatus* (Drew & Romig, 2013). Asia-Pacific. Non-pest. Cue-lure.

*Zeugodacus kaghanae* (Mahmood, 1999). Asia-Pacific. Non-pest. Cue-lure.

*Zeugodacus khaoyaiae* (Drew & Romig, 2013). Asia-Pacific. Non-pest. Cue-lure.

*Zeugodacus laguniensis* (Drew & Romig, 2013). Asia-Pacific. Non-pest. Cue-lure.

*Zeugodacus lipsanus* (Hendel, 1915). Asia-Pacific. Non-pest.

*Zeugodacus liquidus* (Drew & Romig, 2013). Asia-Pacific. Non-pest. Cue-lure.

*Zeugodacus longicaudatus* (Perkins, 1938). Asia-Pacific. Non-pest. Cue-lure.

*Zeugodacus longivittatus* (Chua & Ooi, 1998). Asia-Pacific. Non-pest. Methyl eugenol.

*Zeugodacus luteicinctutus* (Ito, 2011). Asia-Pacific. Non-pest.

Notes: *Z. luteicinctutus* is not included in the Drew and Romig (2013, 2016) keys. According to the diagnosis it is similar to *Z. yoshimotoi*, but differs in having dull brownish instead of shining a black marking surrounding the ocellar triangle. This may prove to be a junior synonym of *Z. yoshimotoi* when more specimens are studied or when molecular data become available.

*Zeugodacus macrophyllae* (Drew & Romig, 2013). Asia-Pacific. Non-pest.

*Zeugodacus macrovittatus* (Drew, 1989). Asia-Pacific. Non-pest. Cue-lure.

*Zeugodacus maculatus* (Perkins, 1938). Asia-Pacific. Non-pest. Cue-lure.

*Zeugodacus maculifacies* (Hardy, 1973). Asia-Pacific. Non-pest. Cue-lure.

*Zeugodacus maculifemur* (Hering, 1938). Asia-Pacific. Non-pest.

*Zeugodacus magnicauda* (White & Evenhuis, 1999). Asia-Pacific. Non-pest.

*Zeugodacus melanofacies* (Drew & Romig, 2013). Asia-Pacific. Non-pest. Cue-lure.

*Zeugodacus melanopsis* (Hardy, 1982). Asia-Pacific. Non-pest. Cue-lure.

*Zeugodacus menglanus* (Yu Liu & Yang, 2011). Asia-Pacific. Non-pest. Cue-lure.

*Zeugodacus mesonotaitha* (Drew, 1989). Asia-Pacific. Non-pest.

*Zeugodacus minimus* (Hering, 1952). Asia-Pacific. Non-pest.

*Zeugodacus montanus* (Hardy, 1983), comb. nov. Asia-Pacific. Non-pest. Cue-lure.

Notes: Originally described in *Dacus*, here transferred from *Bactrocera*. See further comments under *Z. javanensis*.

*Zeugodacus mukiae* (Drew & Romig, 2013). Asia-Pacific. Non-pest. Cue-lure.

*Zeugodacus mundus* (Bezzi, 1919). Asia-Pacific. Cucurbitaceae fruit pest.

*Zeugodacus nakhonnayokiae* (Drew & Romig, 2013). Asia-Pacific. Non-pest. Cue-lure.

*Zeugodacus namlingiae* (Drew & Romig, 2013). Asia-Pacific. Non-pest. Cue-lure.

*Zeugodacus neoelegantulus* (White, 1999). Asia-Pacific. Non-pest.

*Zeugodacus neoemittens* (Drew & Romig, 2013). Asia-Pacific. Non-pest. Cue-lure.

*Zeugodacus neoflavipilosus* (Drew & Romig, 2013). Asia-Pacific. Non-pest. Cue-lure.

*Zeugodacus neolipsanus* (Drew & Romig, 2013). Asia-Pacific. Non-pest. Cue-lure.

*Zeugodacus neopallescentis* (Drew, 1989). Asia-Pacific. Non-pest. Cue-lure.

*Zeugodacus nigrifacies* (Shiraki, 1933). Asia-Pacific. Non-pest.

*Zeugodacus ochrosterna* (Drew & Romig, 2013). Asia-Pacific. Non-pest. Cue-lure.

*Zeugodacus okunii* (Shiraki, 1933). Asia-Pacific. Non-pest.

*Zeugodacus pahangiae* (Drew & Romig, 2013). Asia-Pacific. Non-pest.

*Zeugodacus pantabanganiae* (Drew & Romig, 2013). Asia-Pacific. Non-pest. Cue-lure.

*Zeugodacus papuaensis* (Malloch, 1939), comb. nov. Asia-Pacific. Non-pest.

Notes: This species was moved from *Dacus* to the subgenus Austrodacus by Hancock and Drew (2016), but they continued to classify that subgenus in *Bactrocera*. Like all members of the subgenus Austrodacus, we here place it in the genus *Zeugodacus*.

*Zeugodacus paululus* (Drew, 1989). Asia-Pacific. Non-pest. Cue-lure.

*Zeugodacus pemalangiae* (Drew & Romig, 2013). Asia-Pacific. Non-pest. Cue-lure.

*Zeugodacus perplexus* (Walker, 1862). Asia-Pacific. Non-pest.

*Zeugodacus perpusillus* (Drew, 1971). Asia-Pacific. Non-pest. Cue-lure.

*Zeugodacus persignatus* (Hering, 1941). Asia-Pacific. Non-pest. Cue-lure.

*Zeugodacus platamus* (Hardy, 1973). Asia-Pacific. Non-pest. Cue-lure.

*Zeugodacus proprescutellatus* (Zhang Che & Gao, 2011). Asia-Pacific. Non-pest. Cue-lure.

*Zeugodacus pubescens* (Bezzi, 1919). Asia-Pacific. Non-pest.

*Zeugodacus purus* (White, 1999). Asia-Pacific. Non-pest.

*Zeugodacus quasiinfestus* (Drew & Romig, 2013). Asia-Pacific. Non-pest. Cue-lure.

*Zeugodacus reflexus* (Drew, 1971). Asia-Pacific. Non-pest. Cue-lure.

*Zeugodacus rubellus* (Hardy, 1973). Asia-Pacific. Non-pest.

*Zeugodacus sabahensis* (Drew & Romig, 2013). Asia-Pacific. Non-pest. Cue-lure.

*Zeugodacus sandaracinus* (Drew, 1989). Asia-Pacific. Non-pest.

*Zeugodacus sasaotiae* (Drew & Romig, 2013). Asia-Pacific. Non-pest. Cue-lure.

*Zeugodacus scutellaris* (Bezzi, 1913). Asia-Pacific. Cucurbitaceae flower pest. Cue-lure.

*Zeugodacus scutellarius* (Bezzi, 1916), comb. nov. Asia-Pacific. Non-pest. Cue-lure.

Notes: Originally described in *Chaetodacus*, here transferred from *Bactrocera*. See further comments under *Z. javanensis*.

*Zeugodacus scutellatus* (Hendel, 1912). Asia-Pacific. Cucurbitaceae flower pest. Cue-lure.

*Zeugodacus scutellinus* (Bezzi, 1916). Asia-Pacific. Non-pest.

*Zeugodacus semisurstyli* (Drew & Romig, 2013), comb. nov. Asia-Pacific. Non-pest. Cue-lure.

Notes: Here transferred from *Bactrocera*. See further comments under *Z. javanensis*.

*Zeugodacus semongokensis* (Drew & Romig, 2013). Asia-Pacific. Non-pest. Cue-lure.

*Zeugodacus sepikae* (Drew, 1989). Asia-Pacific. Non-pest.

*Zeugodacus signatifer* (Tryon, 1927). Asia-Pacific. Non-pest.

*Zeugodacus signatus* (Hering, 1941). Asia-Pacific. Non-pest. Cue-lure.

*Zeugodacus sinensis* (Yu Bai & Chen, 2011). Asia-Pacific. Non-pest. Cue-lure.

*Zeugodacus singularis* (Drew, 1989). Asia-Pacific. Non-pest. Cue-lure.

*Zeugodacus sonlaiae* (Drew & Romig, 2013). Asia-Pacific. Non-pest. Cue-lure.

*Zeugodacus speciosus* (Drew & Romig, 2013). Asia-Pacific. Non-pest. Cue-lure.

*Zeugodacus spectabilis* (Drew & Romig, 2013). Asia-Pacific. Non-pest. Cue-lure.

*Zeugodacus strigifinis* (Walker, 1861). Asia-Pacific. Cucurbitaceae flower pest. Cue-lure.

*Zeugodacus sumbensis* (Hering, 1953). Asia-Pacific. Non-pest.

*Zeugodacus surrufulus* (Drew, 1989). Asia-Pacific. Non-pest. Cue-lure.

*Zeugodacus synnephes* (Hendel, 1913). Asia-Pacific. Non-pest. Cue-lure.

*Zeugodacus tapervitta* (Mahmood, 1999). Asia-Pacific. Cucurbitaceae fruit pest.

*Zeugodacus tappanus* (Shiraki, 1933). Asia-Pacific. Non-pest.

*Zeugodacus tau* (Walker, 1849). Asia-Pacific. Cucurbitaceae fruit pest. Cue-lure.

Notes: *Zeugodacus tau* possibly represents a cryptic species complex the extent of which is currently unclear (Baimai 2000, Kitthawee and Dujardin 2010, Kitthawee and Rungsri 2011, Dujardin and Kitthawee 2013).

*Zeugodacus tebeduiae* (Drew & Romig, 2013). Asia-Pacific. Non-pest. Cue-lure.

*Zeugodacus timorensis* (Perkins, 1939). Asia-Pacific. Non-pest. Cue-lure.

*Zeugodacus transversus* (Hardy, 1982). Asia-Pacific. Non-pest. Cue-lure.

*Zeugodacus triangularis* (Drew, 1968). Asia-Pacific. Cucurbitaceae flower pest. Cue-lure, zingerone.

*Zeugodacus trichosanthes* (Drew & Romig, 2013). Asia-Pacific. Cucurbitaceae fruit pest. Cue-lure.

*Zeugodacus trichotus* (May, 1962). Asia-Pacific. Non-pest. Cue-lure.

*Zeugodacus tricuspidatae* (Drew & Romig, 2013). Asia-Pacific. Non-pest.

*Zeugodacus trilineatus* (Hardy, 1955), comb. nov. Asia-Pacific. Non-pest. Cue-lure.

Notes: Originally described in *Dacus*, here transferred from *Bactrocera*. See further comments under *Z. javanensis*.

*Zeugodacus trimaculatus* (Hardy & Adachi, 1954). Asia-Pacific. Cucurbitaceae fruit pest.

*Zeugodacus trivandrumensis* (Drew & Romig, 2013). Asia-Pacific. Non-pest.

*Zeugodacus ujungpandangiae* (Drew & Romig, 2013). Asia-Pacific. Non-pest. Cue-lure.

*Zeugodacus uncinatus* (Drew & Romig, 2013). Asia-Pacific. Non-pest. Cue-lure.

*Zeugodacus unilateralis* (Drew, 1989). Asia-Pacific. Non-pest.

*Zeugodacus univittatus* (Drew, 1972). Asia-Pacific. Non-pest. Cue-lure.

*Zeugodacus urens* (White, 1999). Asia-Pacific. Non-pest.

*Zeugodacus vargus* (Hardy, 1982). Asia-Pacific. Non-pest. Cue-lure.

*Zeugodacus vinnulus* (Hardy, 1973). Asia-Pacific. Non-pest. Cue-lure.

*Zeugodacus vultus* (Hardy, 1973). Asia-Pacific. Non-pest. Cue-lure

*Zeugodacus waimitaliae* (Drew & Romig, 2013). Asia-Pacific. Non-pest. Cue-lure.

*Zeugodacus watersi* (Hardy, 1954). Asia-Pacific. Non-pest.

*Zeugodacus whitei* (Drew & Romig, 2013). Asia-Pacific. Non-pest. Cue-lure.

*Zeugodacus yalaensis* (Drew & Romig, 2013). Asia-Pacific. Non-pest.

*Zeugodacus yoshimotoi* (Hardy, 1973). Asia-Pacific. Non-pest. Cue-lure.

*Zeugodacus zahadi* (Mahmood, 1999). Asia-Pacific. Non-pest. Cue-lure.

Notes: The characters that supposedly distinguish *Z. zahadi* from *Z. tau* overlap, and *Z. zahadi* may be a synonym of *Z. tau* (Drew & Romig, 2013). See further notes under *Z. tau*.

## Supplementary materials

Supplementary material 1 A global checklist of the 932 fruit fly species in the tribe Dacini (Diptera: Tephritidae), spreadsheet table. Data type: Checklist This is an open access article distributed under the terms of the Creative Commons Attribution License (CC BY 4.0), which permits unrestricted use, distribution, and reproduction in any medium, provided the original author and source are credited. Camiel Doorenweerd, Luc Leblanc, Allen L. Norrbom, Michael San Jose, Daniel Rubinoff

## References

[B1] Baimai V (2000) Cytological evidence for a complex of species within the taxon *Bactrocera tau* (Diptera: Tephritidae) in Thailand. Biological Journal of the Linnean Society 69: 399–409. https://doi.org/10.1006/bijl.1999.0377

[B2] CABI (2017) Invasive Species Compendium. http://www.cabi.org/isc/ [accessed Oct. 2017]

[B3] Cameron EC Sved JA Gilchrist AS (2010) Pest fruit fly (Diptera: Tephritidae) in northwestern Australia: One species or two? Bulletin of Entomological Research 100: 197–206. https://doi.org/10.1017/S0007485309990150 10.1017/S0007485309990150 19602297

[B4] Clarke AR Armstrong KF Carmichael AE Milne JR Raghu S Roderick GK Yeates DK (2005) Invasive phytophagous pests arising through a recent tropical evolutionary radiation: the *Bactrocera dorsalis* complex of fruit flies. Annual Review of Entomology 50: 293–319. https://doi.org/10.1146/annurev.ento.50.071803.130428 10.1146/annurev.ento.50.071803.130428 15355242

[B5] David KJ Hancock DL Singh SK Ramani S Behere GT Salini S (2017) New species, new records and updated subgeneric key of *Bactrocera* Macquart (Diptera: Tephritidae: Dacinae: Dacini) from India. Zootaxa 4272: 386–400. https://doi.org/10.11646/zootaxa.4272.3.4 28610282 10.11646/zootaxa.4272.3.4

[B6] David KJ Ramani S Whitmore D Ranganath HR (2016) Two new species and a new record of *Bactrocera* Macquart (Diptera: Tephritidae: Dacinae: Dacini) from India. Zootaxa 4103: 25–34. https://doi.org/10.11646/zootaxa.4103.1.2 10.11646/zootaxa.4103.1.2 27394610

[B7] De Meyer M Delatte H Mwatawala M Quilici S Vayssières JF Virgilio M (2015) A review of the current knowledge on *Zeugodacus cucurbitae* (Coquillett) (Diptera, Tephritidae) in Africa, with a list of species included in *Zeugodacus* ZooKeys 540: 539–557 + Supplementary material S1:(4). https://doi.org/10.3897/zookeys.540.9672 10.3897/zookeys.540.9672 PMC4714087 26798277

[B8] De Meyer M White I (2016) True Fruit Flies (Diptera, Tephritidae) of the Afrotropical Region. http://projects.bebif.be/fruitfly/index.html [accessed Oct. 2017]

[B9] Drew RAI (1971) New species of Dacinae (Diptera: Trypetidae) from the South Pacific Area. Queensland Journal of Agricultural Science 28: 29–103.

[B10] Drew RAI (1989) The tropical fruit flies (Diptera: Tephritidae: Dacinae) of the Australasian and Oceanian Regions. Memoirs of the Queensland Museum 1: 1–536.

[B11] Drew RAI Hancock DL (1994) The *Bactrocera dorsalis* complex of fruit flies (Diptera: Tephritidae: Dacinae) in Asia. Bulletin of Entomological Research Supplement Series 2: 1–68. https://doi.org/10.1017/S1367426900000278

[B12] Drew RAI Hancock DL (2016) A review of the subgenus Bulladacus Drew & Hancock of *Bactrocera* Macquart (Diptera: Tephritidae: Dacinae), with description of two new species from Papua New Guinea. Australian Entomologist 43: 189–210.

[B13] Drew RAI Ma J Smith S Hughes JM (2011) The taxonomy and phylogenetic relationships of species in the *Bactrocera musae* complex of fruit flies (Diptera: Tephritidae: Dacinae) in Papua New Guinea. Raffles Bulletin of Zoology 59: 145–162.

[B14] Drew RAI Romig MC (2013) Tropical fruit flies of South-East Asia. CABI, Wallingford, 655 pp.

[B15] Drew RAI Romig MC (2016) Keys to the Tropical Fruit Flies of South-East Asia. CABI, Wallingford, 487 pp.

[B16] Dujardin JP Kitthawee S (2013) Phenetic structure of two *Bactrocera tau* cryptic species (Diptera: Tephritidae) infesting *Momordica cochinchinensis* (Cucurbitaceae) in Thailand and Laos. Zoology 116: 129–138. https://doi.org/10.1016/j.zool.2012.07.004 23332679 10.1016/j.zool.2012.07.004

[B17] Dupuis JR Bremer FT Kauwe A San Jose M Leblanc L Rubinoff D Geib S (2017) HiMAP: Robust phylogenomics from highly multiplexed amplicon sequencing. bioRxiv. http://biorxiv.org/content/early/2017/11/05/213454.abstract 10.1111/1755-0998.12783 29633537

[B18] Ebina T Ohto K (2006) Morphological characters and PCR-RFLP markers in the interspecific hybrids between *Bactrocera carambolae* and *B. papayae* of the *B. dorsalis* species complex (Diptera: Tephritidae). Research Bulletin of the Plant Protection Service, Japan 42: 23–34.

[B19] Ekesi S De Meyer M Mohamed SA Virgilio M Borgemeister C (2016) Taxonomy, ecology, and management of native and exotic fruit fly species in Africa. Annual Review of Entomology 61: 219–238. https://doi.org/10.1146/annurev-ento-010715-023603 10.1146/annurev-ento-010715-023603 26735644

[B20] Fay HAC (2012) A highly effective and selective male lure for *Bactrocera jarvisi* (Tryon) (Diptera: Tephritidae). Australian Journal of Entomology 51: 189–197. https://doi.org/10.1111/j.1440-6055.2011.00847.x

[B21] Fletcher B (1987) The biology of Dacine fruit flies. Annual Review of Entomology 32: 115–144. https://doi.org/10.1146/annurev.ento.32.1.115

[B22] Freidberg A Kovac D Shiao S (2017) A revision of *Ichneumonopsis* Hardy, 1973 (Diptera: Tephritidae: Dacinae: Gastrozonini) , Oriental bamboo-shoot fruit flies. European Journal of Taxonomy 317: 1–23. https://doi.org/10.5852/ejt.2017.317

[B23] Gilchrist AS Wang Y Yu H Raphael K (2003) Genetic delineation of sibling species of the pest fruit fly *Bactocera* (Diptera: Tephritidae) using microsatellites. Bulletin of Entomological Research 93: 351–360. https://doi.org/10.1079/BER2003249 12908921 10.1079/ber2003249

[B24] Han H-Y Choi D-S Ro K-E (2017) Taxonomy of Korean *Bactrocera* (Diptera: Tephritidae: Dacinae) with review of their biology. Journal of Asia-Pacific Entomology 20: 1321–1332. https://doi.org/10.1016/j.aspen.2017.09.011

[B25] Hancock DL (2015) A new subgenus for six Indo-Australian species of *Bactrocera* Macquart (Diptera: Tephritidae: Dacinae) and subgeneric transfer of four other species. Australian Entomologist 42: 39–44.

[B26] Hancock DL Hamacek EL Lloyd AC Elson-Harris MM (2000) The distribution and host plants of fruit flies (Diptera: Tephritidae) in Australia. Information Series QI99067, Queensland Department of Primary Industries, Brisbane, 75 pp.

[B27] Hancock DL Drew RAI (2006) A revised classification of subgenera and species groups in *Dacus* Fabricius (Diptera: Tephritidae). Instrumenta Biodiversitatis 7: 167–205.

[B28] Hancock DL Drew RAI (2015) A review of the Indo-Australasian subgenus Parazeugodacus Shiraki of *Bactrocera* Macquart (Diptera: Tephritidae: Daciniae). Australian Entomologist 42: 91–104.

[B29] Hancock DL Drew RAI (2017a) A review of the subgenus Javadacus Hardy of *Bactrocera* Macquart (Diptera: Tephritidae: Dacinae). Australian Entomologist 44: 105–112.

[B30] Hancock DL Drew RAI (2017b) A review of the Indo-Australian subgenera Heminotodacus Drew, Paradacus Perkins and Perkinsidacus subgen. n. of Bactrocera Macquart (Diptera: Tephritidae: Dacinae). Australian Entomologist 44: 137–146.

[B31] Hardy DE (1954) The *Dacus* subgenera *Neodacus* and *Gymnodacus* of the world (Diptera, Tephritidae). Proceedings of the Entomological Society of Washington 56: 5–23.

[B32] Hardy DE (1955) A reclassification of the Dacini (Tephritidae-Diptera). Annals of the Entomological Society of America 48: 1–13. https://doi.org/10.1093/aesa/48.6.425

[B33] Hardy DE (1976) Resurrection of *Bactrocera* Macquart and clarification of the type-species, *longicornis* Macquart (Diptera: Tephritidae). Proceedings of the Hawaiian Entomological Society 22: 245–249.

[B34] Hendel FG (1927) Einige neue Bohrfliegen (Trypetidae) aus dem Hamburger Museum. Wiener Entomologische Zeitung 44: 58–65.

[B35] Hendrichs J Vera MT De Meyer M Clarke AR (2015) Resolving cryptic species complexes of major tephritid pests. ZooKeys 540: 5–39. https://doi.org/10.3897/zookeys.540.9656 10.3897/zookeys.540.9656 PMC4714062 26798252

[B36] International Commission on Zoological Nomenclature (1999) International Code of Zoological Nomenclature. Fourth edition. In: Ride WDL et al. (Eds) The International Trust for Zoological Nomenclature, London, 306 pp http://www.nhm.ac.uk/hosted-sites/iczn/code/

[B37] Ito S (2011) Die Bohrfliegen aus Nordost-Nepal (Diptera, Tephritidae). Esakia 51: 1–45.

[B38] Kitthawee S Dujardin JP (2010) The geometric approach to explore the *Bactrocera tau* complex (Diptera: Tephritidae) in Thailand. Zoology 113: 243–249. https://doi.org/10.1016/j.zool.2009.12.002 20817492 10.1016/j.zool.2009.12.002

[B39] Kitthawee S Rungsri N (2011) Differentiation in wing shape in the *Bactrocera tau* (Walker) complex on a single fruit species of Thailand. ScienceAsia 37: 308–313. https://doi.org/10.2306/scienceasia1513-1874.2011.37.308

[B40] Krosch MN Schutze MK Armstrong KF Graham GC Yeates DK Clarke AR (2012) A molecular phylogeny for the Tribe Dacini (Diptera: Tephritidae): systematic and biogeographic implications. Molecular Phylogenetics and Evolution 64: 513–523. https://doi.org/10.1016/j.ympev.2012.05.006 22609822 10.1016/j.ympev.2012.05.006

[B41] Leblanc L San Jose M Rubinoff D (2015a) Description of a new species and new country distibution records of *Bactrocera* (Diptera: Tephritidae: Dacinae) from Cambodia. Zootaxa 4012: 593–600. https://doi.org/10.11646/zootaxa.4012.3.12 26623878 10.11646/zootaxa.4012.3.12

[B42] Leblanc L San Jose M Barr N Rubinoff D (2015b) A phylogenetic assessment of the polyphyletic nature and intraspecific color polymorphism in the *Bactrocera dorsalis* complex (Diptera, Tephritidae). ZooKeys 540: 339–367. https://doi.org/10.3897/zookeys.540.9786 10.3897/zookeys.540.9786 PMC4714077 26798267

[B43] Manrakhan A Daneel JH Beck R Virgilio M Meganck K De Meyer M (2017) Efficacy of trapping systems for monitoring of Afrotropical fruit flies. Journal of Applied Entomology. https://doi.org/10.1111/jen.12373

[B44] Munro HK (1984) A taxonomic treatise on the Dacidae (Tephritoidea, Diptera) of Africa. Entomology Memoirs, Department of Agriculture and Water Supply, Republic of South Africa 61: 1–313.

[B45] Nakahara SMM Muraji M (2008) Phylogenetic analyses of *Bactrocera* fruit flies (Diptera: Tephritidae) based on nucleotide sequences of the mitochondrial COI and COII genes. Research Bulletin of the Plant Protection Service Japan 44: 1–12.

[B46] Norrbom AL Carroll LE Thompson FC White IM Freidberg A (1999) Systematic Database of Names. In: Thompson FC (Ed.) Fruit fly expert identification system and systematic information database. Myia (1998) 9: 65–251, & Diptera Data Dissemination Disk (CD-ROM) (1998) 1.

[B47] Norrbom AL (2004) Updates to Biosystematic Database of World Diptera for Tephritidae through 1999. Diptera Data Dissemination Disk (CD-ROM) 2.

[B48] Pape T Blagoderov V Mostovski MB (2011) Order DIPTERA Linnaeus, 1758. Zootaxa 3148: 222–229.

[B49] Pape T Thompson FC (Eds) (2013) Systema Diptorum, Version 1.5. http://diptera.org/ [accessed on Oct. 2018]

[B50] Pest Management in the Pacific Project (2003) Pacific Fruit Fly Project. https://lrd.spc.int/pacific-fruit-fly [accessed on Oct. 2017]

[B51] Roskov Y Abucay L Orrell T Nicolson D Bailly N Kirk PM Bourgoin T DeWalt RE Decock W De Wever A Nieukerken EJ van Zarucchi J Penev L (2017) Species 2000 & ITIS Catalogue of Life, 2017 Annual Checklist. Digital resource at www.catalogueoflife.org/annual-checklist/2017. Species 2000: Naturalis, Leiden, the Netherlands. http://projects.bebif.be/fruitfly/index.html

[B52] Royer JE (2015) Responses of fruit flies (Tephritidae: Dacinae) to novel male attractants in north Queensland, Australia, and improved lures for some pest species. Austral Entomology 54: 411–426. https://doi.org/10.1111/aen.12141

[B53] Royer JE Agovaua S Bokosou J Kurika K Mararuai A Mayer DG Niangu B (2017) Responses of fruit flies (Diptera: Tephritidae) to new attractants in Papua New Guinea. Austral Entomology. https://doi.org/10.1111/aen.12269

[B54] San Jose M Doorenweerd C Leblanc L Barr N Geib SM Rubinoff D (2018) Incongruence between molecules and morphology: A seven-gene phylogeny of Dacini fruit flies paves the way for reclassification (Diptera: Tephritidae). Molecular Phylogenetics and Evolution. https://doi.org/10.1016/j.ympev.2017.12.001 10.1016/j.ympev.2017.12.001 29224785

[B55] San Jose M Leblanc L Rubinoff D (2013) An evaluation of the species status of *Bactrocera invadens* and the systematics of the *Bactrocera dorsalis* (Diptera: Tephritidae) complex. Annals of the Entomological Society of America 106: 684–694. https://doi.org/10.1603/AN13017

[B56] Schutze MK Aketarawong N Amornsak W Armstrong KF Augustinos AA Barr N Bo W Bourtzis K Boykin LM Cáceres C Cameron SL Chapman TA Chinvinijkul S Chomic A De Meyer M Drosopoulou E Englezou A Ekesi S Gariou-Papalexiou A Geib SM Hailstones D Hasanuzzaman M Haymer D Hee AKW Hendrichs J Jessup A Ji Q Khamis FM Krosch MN Leblanc LUC Mahmood K Malacrida AR Mavragani-Tsipidou P Mwatawala M Nishida R Ono H Reyes J Rubinoff D San Jose M Shelly TE Srikachar S Tan KH Thanaphum S Haq I Vijaysegaran S Wee SL Yesmin F Zacharopoulou A Clarke AR (2015a) Synonymization of key pest species within the *Bactrocera* *dorsalis* species complex (Diptera: Tephritidae): Taxonomic changes based on a review of 20 years of integrative morphological, molecular, cytogenetic, behavioural and chemoecological data. Systematic Entomology 40: 456–471. https://doi.org/10.1111/syen.12113

[B57] Schutze MK Mahmood K Pavasovic A Bo W Newman J Clarke AR Krosch MN Cameron SL (2015b) One and the same: Integrative taxonomic evidence that *Bactrocera invadens* (Diptera: Tephritidae) is the same species as the Oriental fruit fly *Bactrocera dorsalis*. Systematic Entomology 40: 472–486. https://doi.org/10.1111/syen.12114

[B58] Schutze MK Virgilio M Norrbom A Clarke AR (2017) Tephritid integrative taxonomy: Where we are now, with a focus on the resolution of three tropical fruit fly species complexes. Annual Review of Entomology 62: 147–164. https://doi.org/10.1146/annurev-ento-031616-035518 10.1146/annurev-ento-031616-035518 27813666

[B59] Smith PT Kambhampati S Armstrong KA (2003) Phylogenetic relationships among *Bactrocera* species (Diptera: Tephritidae) inferred from mitochondrial DNA sequences. Molecular Phylogenetics and Evolution 26: 8–17. https://doi.org/10.1016/S1055-7903(02)00293-2 12470933 10.1016/s1055-7903(02)00293-2

[B60] Tan KH Nishida R (2000) Mutual reproductive benefits between a wild orchid, *Bulbophyllum patens*, and *Bactrocera* fruit flies via a floral synomone. Journal of Chemical Ecology 26: 533–546. https://doi.org/10.1023/A:1005477926244 10.1023/a:1016277500007 12184394

[B61] Vargas RI Pinero JC Leblanc L (2015) An overview of pest species of *Bactrocera* fruit flies (Diptera: Tephritidae) and the integration of biopesticides with other biological approaches for their management with a focus on the Pacific region. Insects 6: 297–318. https://doi.org/10.3390/insects6020297 26463186 10.3390/insects6020297 PMC4553480

[B62] Virgilio M Jordaens K Verwimp C White IM De Meyer M (2015) Higher phylogeny of frugivorous flies (Diptera, Tephritidae, Dacini): Localised partition conflicts and a novel generic classification. Molecular Phylogenetics and Evolution 85: 171–179. https://doi.org/10.1016/j.ympev.2015.01.007 25681676 10.1016/j.ympev.2015.01.007

[B63] White IM (1999) Morphological features of the Tribe Dacini (Dacinae): Their significance to behavior and classification. In: Aluja M Norrbom AL (Eds) Fruit Flies (Tephritidae): Phylogeny and evolution of Behavior. CRC Press, Boca Raton, 505–534. https://doi.org/10.1201/9781420074468.ch20

[B64] White IM (2006) Taxonomy of the Dacina (Diptera:Tephritidae) of Africa and the Middle East. African Entomology Memoir 2: 1–156.

[B65] White IM Headrick DH Norrbom AL Carroll LE (1999) Glossary. In: Aluja M Norrbom AL (Eds) Fruit flies (Tephritidae): Phylogeny and evolution of behavior. CRC Press, Boca Raton, 881–924. https://doi.org/10.1201/9781420074468.sec8

[B66] White IM Elson-Harris MM (1992) Fruit flies of economic significance. CABI, Wallingford, 601 pp.

[B67] Whitman DW Orsak L Greene E (1988) Spider mimicry in fruit flies (Diptera: Tephritidae): Further experiments on the deterrence of jumping spiders (Araneae: Salticidae) by *Zonosemata vittigera* (Coquillett). Annals of the Entomological Society of America 81: 532–536. http://dx.doi.org/10.1093/aesa/81.3.532

[B68] Yu H Deng Y-L Chen N-Z (2012) A new species of the subgenus Sinodacus from Yunnan, China (Diptera, Tephritidae). Acta Zootaxonomica Sinica 37: 834–836.

[B69] Zhang Z-Q (2011) Animal biodiversity: An outline of higher-level classification and survey of taxonomic richness. Zootaxa 3148: 1–237. 10.11646/zootaxa.3703.1.1 26146682

